# Transcriptional response of rat frontal cortex following acute *In Vivo *exposure to the pyrethroid insecticides permethrin and deltamethrin

**DOI:** 10.1186/1471-2164-9-546

**Published:** 2008-11-18

**Authors:** Joshua A Harrill, Zhen Li, Fred A Wright, Nicholas M Radio, William R Mundy, Rogelio Tornero-Velez, Kevin M Crofton

**Affiliations:** 1 Curriculum in Toxicology, University of North Carolina at Chapel Hill, Chapel Hill, North Carolina, USA; 2 Department of Biostatistics and the Carolina Environmental Bioinformatics Research Center, University of North Carolina at Chapel Hill, Chapel Hill, North Carolina, USA; 3 Neurotoxicology Division, National Health and Environmental Effects Research Laboratory, Office of Research and Development, United State Environmental Protection Agency, Research Triangle Park, North Carolina, USA; 4 Human Exposure and Atmospheric Sciences Division, National Exposure Research Laboratory, Office of Research and Development, United States Environmental Protection Agency, Research Triangle Park, North Carolina, USA

## Abstract

**Background:**

Pyrethroids are neurotoxic pesticides that interact with membrane bound ion channels in neurons and disrupt nerve function. The purpose of this study was to characterize and explore changes in gene expression that occur in the rat frontal cortex, an area of CNS affected by pyrethroids, following an acute low-dose exposure.

**Results:**

Rats were acutely exposed to either deltamethrin (0.3 – 3 mg/kg) or permethrin (1 – 100 mg/kg) followed by collection of cortical tissue at 6 hours. The doses used range from those that cause minimal signs of intoxication at the behavioral level to doses well below apparent no effect levels in the whole animal. A statistical framework based on parallel linear (SAM) and isotonic regression (PIR) methods identified 95 and 53 probe sets as dose-responsive. The PIR analysis was most sensitive for detecting transcripts with changes in expression at the NOAEL dose. A sub-set of genes (*Camk1g*, *Ddc*, *Gpd3*, *c-fos *and *Egr1*) was then confirmed by qRT-PCR and examined in a time course study. Changes in mRNA levels were typically less than 3-fold in magnitude across all components of the study. The responses observed are consistent with pyrethroids producing increased neuronal excitation in the cortex following a low-dose *in vivo *exposure. In addition, Significance Analysis of Function and Expression (SAFE) identified significantly enriched gene categories common for both pyrethroids, including some relating to branching morphogenesis. Exposure of primary cortical cell cultures to both compounds resulted in an increase (~25%) in the number of neurite branch points, supporting the results of the SAFE analysis.

**Conclusion:**

In the present study, pyrethroids induced changes in gene expression in the frontal cortex near the threshold for decreases in ambulatory motor activity *in vivo*. The penalized regression methods performed similarly in detecting dose-dependent changes in gene transcription. Finally, SAFE analysis of gene expression data identified branching morphogenesis as a biological process sensitive to pyrethroids and subsequent *in vitro *experiments confirmed this predicted effect. The novel findings regarding pyrethroid effects on branching morphogenesis indicate these compounds may act as developmental neurotoxicants that affect normal neuronal morphology.

## Background

Pyrethroid insecticides represent a significant percentage of the world insecticide market [1]. This usage results in an increased potential for human exposure. Pyrethroid residues have been detected in sediments from agricultural run-off [2], residential dust samples [3] and child-care centers [4]. Pyrethroid metabolites have also been detected in human urine [5]. A major research focus for pyrethroids is determining if compounds belonging to this chemical class act through the same or similar mechanisms-of-action to produce similar adverse health outcomes [6].

Pyrethroids disrupt nervous system function by interacting with membrane bound ion channels and altering their normal gating kinetics [7]. The primary molecular targets of pyrethroids are neuronal voltage-sensitive sodium channels (VSSCs) [6]. Prolongation of whole-cell Na^+ ^currents has been observed in a variety of cultured nervous system tissues exposed to pyrethroids [8-10]. Furthermore, *in vitro *studies utilizing heterologous expression systems have demonstrated that pyrethroids increase sodium current through VSSC isoforms (Na_v_1.2, Na_v_1.4 & Na_v_1.8), although the complete complement of mammalian VSSCs have not been examined for pyrethroid sensitivity [11-13]. Pyrethroids may also alter the gating kinetics of both neuronal voltage-sensitive Ca^+2 ^(VGCCs) and voltage-sensitive Cl^- ^channels [14-17]. Isoforms of all of the aforementioned molecular targets are expressed in the plasma membrane of mammalian neuronal cells.

Pyrethroids affect nervous system function by producing hyperexcitability in neurons and changing neuronal firing rates [18-21]. Acute manifestations of neurotoxicity on mammalian and insect species result from increased neuronal hyperexcitability driven by the actions of pyrethroids at their molecular target sites, primarily VSSCs [21]. Under normal conditions, neuronal excitation at the membrane results in the activation of intracellular signaling pathways that control the induction of gene expression [22]. In some cases, these transcriptional responses led to persistent adaptive changes in cellular functions (i.e. neuronal plasticity) [23,24]. Neuroactive chemicals that alter firing patterns or disrupt neurotransmission trigger the induction of unique groups of gene transcripts which may in turn impact neuronal function [25-27]. While alterations in neuronal excitability are critical events in the toxiciological mechanism-of-action for pyrethroids, the impact of pyrethroid-induced neuronal hyperexcitability on intracellular signaling pathways and inducible gene-regulatory networks is unknown.

The neuronal substrates that mediate acute pyrethroid neurotoxicity *in vivo *are unknown and it is likely that multiple brain regions are involved. However, previous studies have demonstrated rapid accumulation of pyrethroids in the cortex following an acute oral dose, disruption of cortical neuronal firing patterns both *in vivo *and *in vitro*, and induction of gene products known to be upregulated following neuronal excitation [18,28-32]. Presently, the cortex is one of the few brain regions where pharmacokinetic, electrophysiological and biochemical data are available for pyrethroids. These data provide a significant weight of evidence that this brain region may be sensitive to acute pyrethroid exposures. The present study aims to characterize the effects of acute pyrethroid exposure on gene expression in the cortex.

In the present study Affymetrix GeneChip^® ^microarrays were used to characterize the global transcriptional response of rat frontal cortex following an acute oral exposure to two model pyrethroids: permethrin and deltamethrin. The dose ranges used included doses that cause minimal neurotoxic signs, as well as doses below apparent 'no adverse effect levels' (NOAEL) in *in vivo *behavioral studies of motor function [33]. Differences in potency between the two compounds are due to differing pharmacokinetic profiles and likely varying pharmacodynamic activities [12,28,30,32]. In the present study, low doses were used to minimize any potential transcriptional changes which may be due solely to excessive systemic toxicity at high pyrethroid doses. Primary goals of this study were to: 1) to use a previously established linear regression (SAM) and a novel isotonic (PIR) regression method [34,35] as discovery and prioritization tools for identification of dose-dependent changes in gene transcription, and 2) to compare the performance of these methods, 3) to confirm pyrethroid-sensitive transcriptional changes in a selected sub-set of genes using qRT-PCR, 4) to examine the time course of these changes and 5) to utilize functional category level analysis (SAFE) [36] to identify pyrethroid sensitive cellular processes.

Dose-dependent changes in the transcription of several genes (*Camk1g, Ddc, Gpd3, c-fos *and *Egr1*) were discovered and successfully confirmed. Data from qRT-PCR experiments demonstrated clear qualitative similarities in the transcriptional response produced by both compounds. In addition, based on the SAFE analysis results, the hypothesis that pyrethroids can affect neuronal branching morphology was tested in an *in vitro *model of cortical neuron development. An increase (~25%) in the number of neuronal branch points was observed. This may represent a novel aspect of pyrethroid neurotoxicity that to date has not been examined.

## Methods

### Chemicals

Permethrin (92.0% purity, isomer composition: 40% *cis*, 60% *trans*, 1:1 ratio of 1*R *& 1*S*) and deltamethrin (98.9% purity, isomer composition: 100% 1*R*, 3*R*, α*S*) were generously donated by FMC Corporation (Philadelphia, PA) and Bayer Cropscience (Research Triangle Park, NC), respectively. Pyrethroids were dissolved in corn oil (Sigma-Aldrich, St. Louis, MO) at 1, 10, 40 & 100 mg/mL permethrin and 0.3, 1 & 3 mg/mL deltamethrin. Dosing volume was 1 mL/kg.

### Animal Care and Treatment

Male Long-Evans rats (49–62 days of age) were obtained from Charles River Laboratories (Wilmington, MA) Housing conditions were identical to those used in Wolansky et al. (2006) [33]. The facility was approved by the American Association for Accreditation of Laboratory Animal Care (AAALAC) and all experimental procedures were approved in advance by the US EPA, NHEERL Animal Care and Use Committee.

Four cohorts of animals were used in this study (Table 1). Cohort 1 was used for preliminary data collection to demonstrate that the selected doses of the two compounds would alter gene transcription. Cohort 2 replicated these findings and expanded group sizes. Cohorts 1 & 2 were combined for microarray data analyses. Cohort 3 was examined exclusively by quantitative real-time RT-PCR (qRT-PCR). These dose-response cohorts (#1,2,3) were exposed on separate days. All dosing occurred between 06:30 and 07:00 hours, counterbalanced across time of day, and the last test subject euthanized before 18:00 hours. Cohort 4 was used in qRT-PCR time course studies and dosed with 3 mg/kg deltamethrin, 100 mg/kg permethrin or vehicle. Each time point contained pyrethroid-treated and time-matched vehicle controls and all test subjects were dosed and euthanized between 07:30 & 17:30 hours. In all cohorts, test subjects were removed from the colony suite 1 h prior to dosing and allowed to acclimate in a quiet holding room maintained under similar environmental conditions. Subjects were administered a single oral dose of test compound by gavage and allowed to recover in their home cage prior to euthanasia at 6 h (dose-response studies) or 1, 3, 6 or 9 h (time course studies). Subjects were removed to an adjoining suite with a separate HVAC system for euthanasia by decapitation. Whole brains were rapidly removed onto a cold plate (4°C). Frontal cortex was dissected and frozen on a bed of dry ice in less than 3 minutes and then stored at -80°C until use.

**Table 1:** Group sizes of cohorts used in this study.

		**Permethrin**				**Deltamethrin**		
**Dose (mg/kg):**	**Vehicle**	**1.0**	**10.0**	**40.0**	**100.0**	**0.3**	**1.0**	**3.0**
**EDL:**	**Control**	**< NOAEL**	**NOAEL**	**ED**_30_	**ED**_50_	**< NOAEL**	**NOAEL**	**ED**_30_
**Microarray Dose Response **^**a**^								
**Cohort 1**	6	3	3		3	3	3	3
**Cohort 2**	6	5	5		5	5	5	5
**qRT-PCR Dose-Response **^**b**^								
**Cohort 3**	7	7	7	7	7			
	7					7	7	7
**qRT-PCR Time Course **^**c**^								
**Cohort 4**	8_4_				8_4_			
	8_4_							8_4_

^a ^Microarray data from Cohorts 1 & 2 were combined (n = 8/treatment group) with control values from cohorts 1 & 2 (n = 12) for dose-response analysis of permethrin and deltamethrin mediated effects, respectively. ^b ^Test animals in Cohort 3 were split into two dose-response studies for permethrin and deltamethrin, respectively, for qRT-PCR confirmation of gene expression changes observed during the microarray study. ^c ^Test animals in Cohort 4 were used for qRT-PCR time course studies. Four time points (1,3,6,9 hours: n = 8/treatment group) per compound with time matched controls (n = 8/control group). NOAEL = no observable adverse effect level; < NOAEL = less than no observable adverse effect level.

### RNA Extraction

Cortical samples were homogenized in 1 mL of TRI Reagent (Molecular Research Center, Inc., Cincinnati, OH) per 50–100 mg of tissue using a Polytron^® ^PT-K homogenizer (Kinematica, Lucerne, Switzerland) and total RNA was isolated per manufacturer's instructions. Total RNA pellets suspended in DEPC-treated H_2_O were then subject to DNase I treatment and re-extracted with acid:phenol chloroform, pH = 4.7 (Ambion Inc., Austin, TX) and chloroform according to manufacturer's protocol and re-suspended in DEPC-treated H_2_O until use. The total RNA concentration of each sample was determined (absorbance @ 260 nm) on a Beckman-Coulter DU^® ^800 spectrophotometer (Fullerton, CA) and adjusted to 1.0 μg/μL prior to sample storage at -80°C. The ratio of absorbance values at 260 nm and 280 nm (Ab 260/280) was used to assess purity of total RNA samples and a cut-off of > 1.6 was used (greater than 85% of the samples were > 1.7). Preliminary PCR experiments using primers for rat β-actin genomic DNA (outlined in [37]) demonstrated that the above protocol adequately prevents genomic DNA contamination of total RNA samples (data not shown). RNA integrity of each sample was determined using an Agilent 2100 Bioanalyzer and RNA 6000 Nano LabChip Kit (Waldbron, Germany) according to manufacturer's instructions. All samples used in microarray and qRT-PCR experiments had a RNA Integrity Number (RIN) > 8.0 (data not shown). Aliquots of each RNA sample (1 μg/μL for microarray hybridization or 25 ng/μL for qRT-PCR assays) were stored at -80°C until use.

### Microarray sample preparation and data collection

All protocols for microarray sample preparation (except total RNA extraction, as above), Affymetrix Rat Genome 230 2.0 GeneChip^® ^hybridization, array scanning and data collection were performed by Expression Analysis, Inc., (Durham, NC) according to standard Affymetrix protocols. Synthesis and clean-up of biotin-labeled cRNA was performed using a BioArray™ High Yield™ RNA transcript labeling kit (Enzo Life Sciences, Farmingdale, NY) and Qiagen RNeasy spin columns (Spoorstraat, Netherlands), respectively, according to manufacturer's instructions. Hybridizations were performed in an Affymetrix Hybridization Oven 640. Washes were performed on an Affymetrix Fluidics Station 450 using the EukGE-WS2v4-450 fluidics script. GeneChips^® ^were scanned using an Affymetrix GeneChip^® ^3000 Scanner with the GCOS v1.2 software package. Target intensity was set to a value of 500 with all other scanning parameters set at the factory defaults. The 3'/5' ratios for GAPDH and β-actin internal controls genes ranged between 0.93 – 1.11 and 1.2 – 2.01, respectively for all samples, indicating that degradation of RNA did not occur. The intensity of hybridization controls (*BioB*, *BioC*, *BioD *and *Cre*) increased linearly on all arrays. Gene expression profiles for this experiment have been archived in the NCBI Gene Expression Omnibus (GEO) repository with the series accession number GSE7955.

### Microarray Data Analysis

Expression summaries were calculated using RMAExpress^© ^v4.7 (University of California at Berkeley). Consistent with previous reports, Robust Multiarray Average (RMA) [38] provided less within group variation in expression summary values as compared to GeneChip^® ^Operating Software v1.2 (GCOS) [39] (see Additional file 1).

Analysis of dose-response relationships were performed using Significance Analysis of Microarrays (SAM, version 2.21) [34], with the quantitative/linear regression modeling component [40]. In addition to identifying dose-responsive genes, SAM provides permutation-based estimates of the false-discovery rate (*FDR*) associated with lists of genes for which the null hypothesis is rejected. The SAM statistic (*d*_*i*_) penalizes lowly expressed genes, and is most powerful when the dose-response function is nearly linear in the range examined. To potentially increase power and account for non-linearity in dose-response relationships, the SAM analyses were supplemented by penalized isotonic regression (PIR) according to the method of Hu et al. (2005) [35] which was specifically designed for microarray analysis. Similar to SAM, PIR penalizes lowly expressed genes and provides a permutation-based estimate of the false discovery rate. In contrast to SAM, PIR allows for the dose-response relationship to be nonlinear, but assumes the relationship is increasing or decreasing as a function of increasing dose, and not the reverse direction. This method results in a score (the *M*_*i*_-statistic) for each probe set that quantifies the evidence for an increasing or decreasing dose-response relationship.

To insure that the rigorously conservative, permutation-based approaches for controlling Type I error did not exclude true positive probe sets with dose-dependent increases or decreases in expression, an additional analysis was conducted with each regression model. Empirical *p*-values from the PIR analysis or SAM analysis were used to filter out probe sets with no apparent dose-related changes in expression (threshold *p*-value < 0.01). The reduced group of probe sets were then analyzed using a one-way analysis of variance (ANOVA) followed by a Benjamini-Hochberg correction for control of multiple comparisons. Dose was used as the independent factor. Probe sets meeting the Benjamini-Hochberg correction at *FDR *< 0.05 were included in the gene lists of interest for each compound, analysis of dose thresholds for transcriptional changes and the comparison of effects between compounds. For probe sets that passed the one-way ANOVA significance threshold, a post-hoc Dunnett's multiple-comparison mean contrast test was performed comparing the means of the respective dose groups to the mean of the control group [41]. Regression analyses were performed using R^© ^version 2.3.0 statistical computing analysis software. Dunnett's tests were performed using SAS v8.1 (SAS Institute, Inc., Cary, NC).

### Quantitative real-time RT-PCR

In selecting candidates for qRT-PCR confirmation, preference was given to probe sets highly ranked by the penalized regression methods and corresponding to transcripts with known protein-coding RefSeq accession numbers (Tables 2 &3). qRT-PCR for each transcript of interest was performed using TaqMan^® ^One-Step RT-PCR Master Mix Reagent Kits and TaqMan^® ^Gene Expression Assays on an ABI 7900HT Sequence Detection System (Applied Biosystems, Foster City, CA) according to manufacturer's instructions and using a 384 well plate format. Each sample was measured in triplicate for each transcript of interest and an internal reference gene. Reaction plates were maintained at 5°C during the loading procedure. Reactions were incubated at 48°C for 45 min followed by incubation at 95°C for 10 min and 40 cycles of 94°C for 25 sec followed by 60°C for 1 min. qRT-PCR assays were designed via the Applied Biosystems (ABI) primer/probe selection algorithm and bioinformatics pipeline [42]. Amplification efficiencies for each assay was calculated as previously described using a serial dilution of pooled total RNA from rat frontal cortex [43]. Assay identification numbers, context sequences, amplicon lengths and calculated amplification efficiencies are listed in Additional file 2.

**Table 2:** Probe sets identified as dose-responsive for deltamethrin.

		Linear Regression (SAM)			Isotonic Regression (PIR)			ANOVA
Affymetrix Probe Set I.D.	Gene Symbol	*d*_*i*_	*p*-value	*q*-value	*M*_*i*_	*p*-value	*q*-value	*p*-value
1371363_at	Gpd1**	5.63	0.0000	0.00	2.34	0.0000	0.24	0.0032
1388901_at	Fkbp51**	5.46	0.0000	0.00	2.21	0.0000	0.42	0.0051
1367577_at	Hsp27**	4.60	0.0001	0.00	1.90	0.0002	0.97	0.0195
1369560_at	Gpd1**	4.53	0.0001	0.00	1.91	0.0002	1.00	0.0134
1368064_a_at	Ddc**	-4.35	0.0001	0.14	-1.95	0.0002	1.00	0.0195
1391229_at	Camk1g**	4.22	0.0002	0.00	2.11	0.0000	0.51	0.0063
1380611_at	Fkbp51**	3.90	0.0004	0.11	1.62	0.0012	1.00	0.0276
1369303_at	*Crh*	3.62	0.0009	0.11	1.73	0.0006	1.00	0.0276
1388271_at	*LOC689415*	3.48	0.0013	0.19	1.44	0.0037	1.00	0.0300
1370989_at	*Ret*	3.38	0.0017	0.19	1.49	0.0028	1.00	0.0300
1370026_at	*Cryab*	3.28	0.0022	0.29	1.46	0.0032	1.00	0.0364
1374626_at	*Lrg1*	3.23	0.0025	0.33	1.45	0.0036	1.00	0.0323
1376709_at	*Slc39a8*	-3.07	0.0038	0.14	-1.49	0.0026	1.00	0.0364
1368650_at	*Klf10*	-3.07	0.0038	0.14	-1.30	0.0077	1.00	0.0356
1380329_at	*Tmem10*	-3.03	0.0041	0.14	-1.28	0.0086	1.00	0.0496
1372564_at	*Ets2*	3.03	0.0042	0.51	1.41	0.0045	1.00	0.0396
1389507_at	*Nedd4l*	2.97	0.0048	0.51	1.42	0.0043	1.00	0.0306
1375138_at	*Timp3*	2.92	0.0054	0.56	1.22	0.0134	1.00	0.0319
1370530_a_at	*Pld1*	-2.81	0.0071	0.14	-1.34	0.0062	1.00	0.0493
1385778_at	*Siat7E*	2.79	0.0075	1.15	1.38	0.0055	1.00	0.0276
1395986_at	*Slit2*	-2.78	0.0077	0.14	-1.27	0.0094	1.00	0.0419
1369973_at	*Xdh*	2.76	0.0080	1.15	1.18	0.0167	1.00	0.0472
1368438_at	*Pde10a*	2.70	0.0094	1.16	1.11	0.0255	1.00	0.0496
1387260_at	*Klf4*	-2.69	0.0096	0.14	-1.49	0.0026	1.00	0.0429
1372356_at	*Usp54*	2.69	0.0097	1.16	1.12	0.0240	1.00	0.0442
1371442_at	*Hyou1*	2.68	0.0099	1.16	1.03	0.0384	1.00	0.0467
1375296_at	*LOC684097*	2.66	0.0105	1.16	1.40	0.0047	1.00	0.0427
1398899_at	*Polr2c*	2.64	0.0110	1.16	1.67	0.0009	1.00	0.0195
1377518_at	Camk1g**	2.59	0.0123	1.16	1.29	0.0090	1.00	0.0315
1372090_at	*Max*	2.53	0.0143	1.16	1.31	0.0080	1.00	0.0306
1381557_at	*Gna14*	2.35	0.0216	1.16	1.29	0.0087	1.00	0.0442
1398373_at	*B3galt3*	2.29	0.0248	1.16	1.29	0.0091	1.00	0.0396
1372037_at	*Pdlm7*	2.18	0.0319	1.16	1.28	0.0098	1.00	0.0351
1382112_at	*LOC682926*	-2.16	0.0333	1.15	-1.26	0.0096	1.00	0.0344
1375752_at	*Bves*	-1.96	0.0519	1.15	-1.41	0.0042	1.00	0.0376
1370869_at	*Bcat1*	1.92	0.0561	1.16	1.28	0.0096	1.00	0.0345
1367706_at	*Vdac1*	1.74	0.0820	1.16	1.29	0.0090	1.00	0.0295

** = genes examined by qRT-PCR. Positive SAM *d*_*i *_or PIR *M*_*i *_scores denote upregulated probe sets. Negative SAM *d*_*i *_or PIR *M*_*i *_scores denote downregulated probe sets. A full list of probe sets altered by deltamethrin exposure, including predicted protein coding sequences and expressed sequence tags is given in Additional file 3.

**Table 3:** Probe sets identified as dose-responsive for permethrin.

		Linear Regression (SAM)			Isotonic Regression (PIR)			ANOVA
Affymetrix Probe Set I.D.	Gene Symbol	*d*_*i*_	*p*-value	*q*-value	*M*_*i*_	*p*-value	*q*-value	*p*-value
1369303_at	*Crh*	4.26	0.0001	0.00	1.92	0.0003	1.00	0.0113
1368677_at	Bdnf**	3.85	0.0003	0.00	1.52	0.0025	1.00	0.0170
1370412_at	*Slc40a1*	-3.50	0.0006	0.18	-1.70	0.0016	1.00	0.0050
1370415_at	Rassf5**	3.37	0.0008	0.12	1.85	0.0004	1.00	0.0170
1375043_at	c-fos**	3.23	0.0012	0.18	1.41	0.0045	1.00	0.0170
1395991_at	*Rimbp2*	3.11	0.0016	0.28	1.39	0.0050	1.00	0.0400
1388583_at	*Cxcl12*	2.92	0.0027	0.42	1.36	0.0057	1.00	0.0477
1368321_at	Egr1**	2.87	0.0030	0.50	1.21	0.0132	1.00	0.0230
1369067_at	*Nr4a3*	2.77	0.0040	0.55	1.15	0.0187	1.00	0.0270
1387025_at	*Dync1i1*	2.76	0.0041	0.55	1.29	0.0085	1.00	0.0208
1381557_at	*Gna14*	2.67	0.0052	0.61	1.87	0.0004	1.00	0.0176
1387024_at	*Dusp6*	2.59	0.0064	0.75	1.01	0.0398	1.00	0.0230
1388911_at	*Prim2*	2.50	0.0082	0.79	1.45	0.0036	1.00	0.0408
1385778_at	*Siat7E*	2.36	0.0116	0.79	1.43	0.0041	1.00	0.0305
1395272_at	*LOC682937*	-1.88	0.0394	0.69	-1.33	0.0084	1.00	0.0305
1367652_at	*Igfbp3*	1.38	0.1190	0.79	1.35	0.0063	1.00	0.0358
1389090_at	*Wrnip1*	1.32	0.1319	0.79	1.30	0.0079	1.00	0.0170
1376602_a_at	*Fbxo22*	0.89	0.2974	1.32	1.27	0.0096	1.00	0.0186
1391301_at	*LOC682355*	0.51	0.5404	1.32	1.35	0.0062	1.00	0.0305

** = genes examined by qRT-PCR. Positive SAM *d*_*i *_or PIR *M*_*i *_scores denote upregulated probe sets. Negative SAM *d*_*i *_or PIR *M*_*i *_scores denote downregulated probe sets. A full list of probe sets altered by deltamethrin exposure, including predicted protein coding sequences and expressed sequence tags is given in Additional file 4.

qRT-PCR data from deltamethrin and permethrin dose-response and time course studies were analyzed according to the 2^-ΔΔC^_T _method as described by Livak and Schmittgen (2001) [44]. β-actin expression did not change as a function of time or dose for either compound (data not shown) and was used at the internal reference for all 2^-ΔΔC^_T _calculations. Data are expressed as 2^-ΔΔC^_T _± standard error (SE) which is an approximation of fold-change from the calibrator group (i.e. vehicle control). For dose-response studies (Table 4), the mean ΔΔ^C^_T _of vehicle treated controls were used as the 2^-ΔΔC^_T _calibrator [44]. For time course studies, the mean ΔΔ^C^_T _of vehicle treated controls were used as the 2^-ΔΔC^_T _calibrator within each time-matched treatment group.

**Table 4:** qRT-PCR confirmation of transcripts identified as dose-responsive.

	**Deltamethrin**			**Permethrin**		
***Camk1g***^*a*^	***qRT-PCR***	***1391229_at***	***1377518_at***	***qRT-PCR***	***1391229_at***	***1377518_at***
Control	1.03 ± 0.10	*1.00 ± 0.04*	*1.00 ± 0.05*	1.04 ± 0.13	*1.00 ± 0.04*	*1.00 ± 0.05*
< NOAEL	1.23 ± 0.18	*1.10 ± 0.03*	*1.07 ± 0.08*	1.05 ± 0.14	*1.16 ± 0.10*	*1.20 ± 0.13*
NOAEL	1.40 ± 0.18	*1.26 ± 0.07*	*1.31 ± 0.06*	1.97 ± 0.28*	*1.09 ± 0.05*	*1.09 ± 0.09*
ED_30_	1.57 ± 0.14*	*1.31 ± 0.06*	*1.39 ± 0.06*	1.76 ± 0.32*		
ED_50_				1.72 ± 0.30*	*1.25 ± 0.09*	*1.45 ± 0.17*
						
***Ddc ***^*a*^	***qRT-PCR***	***1368064_a_at***		***qRT-PCR***	***1368064_a_at***	
Control	1.01 ± 0.06	*1.00 ± 0.03*		1.04 ± 0.12	*1.00 ± 0.03*	
< NOAEL	0.79 ± 0.06	*0.96 ± 0.04*		0.97 ± 0.10	*1.04 ± 0.04*	
NOAEL	0.89 ± 0.07	*0.90 ± 0.05*		0.91 ± 0.10	*1.01 ± 0.04*	
ED_30_	0.70 ± 0.05*	*0.80 ± 0.03*		0.81 ± 0.11*		
ED_50_				0.71 ± 0.09*	*1.00 ± 0.05*	
						
***Gpd1***^*b*^	***qRT-PCR***	***1371363_at***	***1369560_at***	***qRT-PCR***	***1371363_at***	***1369560_at***
Control	1.06 ± 0.13	*1.00 ± 0.11*	*1.00 ± 0.08*	1.04 ± 0.14	*1.00 ± 0.11*	*1.00 ± 0.08*
< NOAEL	1.16 ± 0.13	*0.88 ± 0.07*	*0.88 ± 0.05*	0.94 ± 0.07	*1.03 ± 0.10*	*0.98 ± 0.09*
NOAEL	1.04 ± 0.12	*1.42 ± 0.17*	*1.25 ± 0.13*	0.97 ± 0.06	*1.03 ± 0.09*	*0.95 ± 0.10*
ED_30_	2.04 ± 0.28*	*1.94 ± 0.19*	*1.55 ± 0.14*	1.24 ± 0.15		
ED_50_				1.23 ± 0.13	*1.19 ± 0.18*	*1.12 ± 0.16*
						
***Fkbp51 ***^*b*^	***qRT-PCR***	***1388901_at***	***1380611_at***	***qRT-PCR***	***1388901_at***	***1380611_at***
Control	1.01 ± 0.06	*1.00 ± 0.05*	*1.00 ± 0.04*	1.02 ± 0.09	*1.00 ± 0.05*	*1.00 ± 0.04*
< NOAEL	0.92 ± 0.06	*1.00 ± 0.03*	*1.04 ± 0.07*	1.03 ± 0.09	*0.92 ± 0.05*	*0.96 ± 0.07*
NOAEL	1.02 ± 0.07	*1.17 ± 0.05*	*1.16 ± 0.08*	1.00 ± 0.06	*1.06 ± 0.03*	*0.99 ± 0.06*
ED_30_	1.52 ± 0.14*	*1.41 ± 0.09*	*1.35 ± 0.10*	0.95 ± 0.07		
ED_50_				1.03 ± 0.12	*1.07 ± 0.05*	*1.07 ± 0.09*
						
***c-fos ***^*c*^	***qRT-PCR***	***1375043_at***		***qRT-PCR***	***1375043_at***	
Control	1.19 ± 0.27	*1.00 ± 0.16*		1.09 ± 0.21	*1.00 ± 0.16*	
< NOAEL	0.63 ± 0.14	*1.01 ± 0.31*		1.49 ± 0.25	*0.54 ± 0.07*	
NOAEL	1.09 ± 0.43	*0.72 ± 0.12*		1.30 ± 0.22	*0.57 ± 0.08*	
ED_30_	0.54 ± 0.08*	*0.69 ± 0.06*		0.91 ± 0.11		
ED_50_				1.25 ± 0.27	*1.56 ± 0.34*	
						
***Egr1 ***^*c*^	***qRT-PCR***	***1368321_at***		***qRT-PCR***	***1368321_at***	
Control	1.04 ± 0.12	*1.00 ± 0.08*		1.01 ± 0.07	*1.00 ± 0.16*	
< NOAEL	0.81 ± 0.05	*0.93 ± 0.09*		1.10 ± 0.10	*0.77 ± 0.02*	
NOAEL	0.90 ± 0.16	*0.95 ± 0.08*		1.10 ± 0.11	*0.89 ± 0.09*	
ED_30_	0.87 ± 0.07	*0.98 ± 0.06*		1.09 ± 0.08		
ED_50_				1.12 ± 0.10	*1.19 ± 0.08*	

Values in normal type-set are 2^-ΔΔCT ^(± SE) from qRT-PCR Cohort 3. For comparison, values in italics are fold-change from control (± SE) for microarray probe sets (Cohorts 1 & 2). Groups for statistical analysis are given in Table 1. Equipotent dose groups are based on Wolansky et al (2006). Main effects in two-way ANOVA: ^a ^= EDL (*p *< 0.05), ^b ^= COMPOUND*EDL (*p *< 0.05) and DOSE (p < 0.05) for deltamethrin only in one-way ANOVA, ^c ^= COMPOUND (*p *< 0.05). * = Significance difference from control in a Dunnett's mean contrast test (*p *< 0.05).

Data from Wolansky et al. (2006) [33] were used to assign equipotent dose-levels (EDL) to the administered doses used in the present study to provide a comparative dose-metric between the two test compounds (see Table 1). Statistical analysis of qRT-PCR dose response data was performed using a two-way ANOVA with compound and equipotent dose level (EDL) as independent variables and 2^-ΔΔC^_T _as the dependent variable followed by Dunnett's mean contrast test. Transcripts with a significant compound by EDL interaction were further analyzed using a one-way ANOVA with dose as the independent variable followed by Dunnett's mean contrast test. Statistical analysis of time course data was performed using a two-way ANOVA with time and treatment as independent variables and 2^-ΔΔC^_T _as the dependent variable. Transcripts with a significant time*treatment interaction (*p *< 0.05) were additionally analyzed with a one-way ANOVA at each time point with treatment as the independent variable (*p *< 0.05).

### Significance Analysis of Function and Expression (SAFE)

The SAFE method was used to identify pathways/functional categories whose genes are coordinately regulated in a dose-dependent manner [36]. SAFE is similar to other pathway enrichment procedures (e.g. DAVID) [45], but accounts for correlation in gene expression within pathways using array permutation to rigorously control error rates. SAFE and accompanying array annotation were loaded from Bioconductor v.1.8 [46]. SAFE tests for enrichment of significant dose-response relationships for genes within each pathway. Following calculation of linear regression dose-response *p*-values for each gene, only genes with a nominal *p*-value < 0.05 were used to form the gene list to which the enrichment analysis was performed. SAFE [47] enables the user to define a pathway enrichment statistic and a Pearson test of binomial proportions was then implemented [48]. The Pearson statistics is similar to Fisher's exact test commonly employed in pathway enrichment testing (GSEA) [49], but does not consider the number of significant genes to have been fixed *a priori *[50]. 10,000 permutations of dose levels were used by SAFE to assess the significance of the entire procedure, using the Yekutieli and Benjamini (1999) procedure to control the *FDR *while accounting for the multiple pathways/categories [51]. All categories with an estimated *FDR *< 0.1 are reported in Table 5.

**Table 5:** Significant Analysis of Function and Expression (SAFE) results.

**Commonly enriched gene categories for both permethrin and deltamethrin**^**a**^			
**Category I.D. and name**	**size**^**b**^	**DLT *p*-value**	**PERM *p*-value**
***GO Biological Process***			
GO:0048754, 'branching morophogenesis of a tube'	66	0.0171	2.00E-04
GO:0001763, 'morphogenesis of a branching structure'	67	0.0172	2.00E-04
GO:0007162, 'negative regulation of cell adhesion'	27	0.0175	0.0025
GO:0015718, 'monocarboxylic acid transport'	30	0.0051	0.0125
GO:0007498, 'mesoderm development'	57	0.0105	0.0067
			
***GO Cellular Component***			
GO:0005954, 'Ca^+2 ^and calmodulin-dependent protein kinase complex'	25	0.0053	0.0146
			
***GO Molecular Function***			
GO:0046915, 'transition metal ion transporter activity'	44	0.0026	0.0348
			
**Enriched gene categories identified by SAFE**.			
**Category I.D. and name**	**size**^**b**^	***p*-value**^**c**^	

**Deltamethrin**			
***KEGG Pathway***			
KEGG:00564, 'Glycerophospholipid metabolism'	73	0.0404	
KEGG:00400, 'Phenylalanine, tyrosine and tryptophan biosynthesis	12	0.0928	
			
**Permethrin**			
***GO Biological Process***			
GO:0048754, 'branching morphogenesis of a tube'	66	0.0349	
GO:0001763, 'morphogenesis of a branching structure'	67	0.0349	
GO:0001569, 'patterning of blood vessels'	31	0.0406	
GO:0009880, 'embryonic pattern specification'	49	0.0554	
GO:0045655, 'regulation of monocyte differentiation'	32	0.0932	

^a ^GO catergories or KEGG pathways with *p *< 0.05 for both test compounds using Fisher's combined *p*-value method to test for joint enrichment in the SAFE procedure. ^b ^number of Affymetrix probe sets included in GO category or KEGG pathway groupings. ^c ^GO categories or KEGG pathways with an adjusted *p *< 0.1 for SAFE method. 700 GO-BP (biological processes), 142 GO-CC (cellular component), 307 GO-MF (molecular function) and 126 KEGG pathways were examined.

### Combining pathway evidence for the two pyrethroids

One aim of using the SAFE statistical methods in this study was to identify gene categories showing enrichment for dose-responsiveness for both permethrin and deltamethrin. The Fisher combined *p*-value method allows accrual of evidence across multiple hypotheses, and thus is ideal for testing combined evidence for enrichment of each pathway for both chemicals [52]. Under the null hypothesis that neither chemical shows enrichment for the pathway, each of the two *p*-values is uniform [0,1], and the Fisher statistic

S = -2(ln(*p*_*delta*_)+ln(*p*_*norm*_))

is distributed as $\chi_{2}^{2}$. The Fisher approach has favorable optimality properties [53] and results in a new (combined) *p*-value for each pathway. For the multiple pathways tested, the Benjamini-Hochberg (1995) method was applied to control the false discovery rate (*FDR *< 0.1) [54].

Fisher's statistic can be asymmetrically sensitive to very small *p*-values for a single chemical, even if the results for the other chemical are not significant. Thus, among pathways with a significant Fisher statistic, the focus was placed on those which showed SAFE *p*-values < 0.05 for both chemicals.

### Cell culture and treatment

Cortical cultures containing neurons and glia were prepared from neocortices of newborn rat pups according to the protocol used by Chandler et al. (1993) with modifications [55]. Neocortices were harvested under sterile conditions in a buffer solution containing 137 mM NaCl, 5 mM KCl, 170 μM Na_2_HPO_4_, 205 μM KH_2_PO_4_, 5 mM glucose, 59 mM sucrose, 100 U/ml penicillin and 0.1 mg/ml streptomycin, pH 7.4. The cortices were minced with scissors and digested using 0.25% trypsin for 5 minutes, then with addition of 0.016% DNase for a further 5 minutes at 37°C and mixed at 30 rpm. The cortices were centrifuged (400 × g, 1600 rpm) for 5 minutes at room temperature, the supernatant was aspirated and the tissue pellet was re-suspended in Gibco^® ^DMEM/GlutaMAX-1 (Invitrogen Corp, Carlsbad, CA) containing 10 mM HEPES, 100 U/ml penicillin, 0.1 mg/ml streptomycin and 10% horse serum, pH = 7.4. The tissue was dissociated by trituration and filtered through a 100-μm Nitex screen. Cells were plated at a density of 50,000 cells/well in 96-well polystyrene plates (Corning, Inc., Corning, NY) that had been pre-coated with poly-L-lysine. Cells were incubated at 37°C in a humidified atmosphere of 5% CO_2 _and 95% air.

Multi-compartment pharmacokinetic models for the disposition of deltamethrin and permethrin were used to predict tissue concentrations of deltamethrin and permethrin in the brain at 6 h following the acute administered doses used in this study [56,57]. Predictions are listed in Table 6. These estimated brain concentrations were then used to select nominal media concentrations of pyrethroids for use in the functional neurite morphogenesis cell model.

**Table 6:** Pharmacokinetic estimates of pyrethroid brain concentrations.

	Administered Dose (mg/kg)	Time (h)	Brain Concentration (μM)
**Deltamethrin**^**a**^	0.3	6	0.005
	1	6	0.0169
	3	6	0.050
			
**Permethrin**^**b**^	1	6	0.060
	10	6	0.582
	100	6	5.940

^a ^Estimates based on Mirfazaelian et al. (2006).

^b ^Estimates based on Tornero-Velez et al. (2007).

For *in vitro *exposure of cells, pyrethroids were prepared in DMSO using semi-logarithmic serial dilutions of concentrated stock solutions to yield final chemical concentration ranges of 0.001 – 0.03 μM and 0.01 – 3 μM for deltamethrin and permethrin, respectively. The final DMSO concentration in the cortical media was 0.1%. Chemicals were added to the cells 2 hours after plating to ensure the cells adhered to the poly-L-lysine and incubated for a 96-hour exposure period.

### Evaluation of neurite outgrowth and cell viability

Immunocytochemical staining with a Neurite Outgrowth Hitkit (Thermo-Fisher Scientific, Waltham, MA) and subsequent analysis using a Cellomics ArrayScan V^TI ^high content imaging platform was used to evaluate cortical cell neurite outgrowth and branching as described in Radio et al. (2008) for differentiated PC-12 cells [58]. The Cellomics ArrayScan V^TI ^Neuronal Profiling Bioapplication used a 10X objective and sampled a sufficient number of fields for the analysis of at least 200 cells per well. Data represent the mean ± standard error across 3 replicate experiments. Cellular viability was determined in cortical cell cultures grown as described above in opaque 96-well plates using the CellTiter-Glo Viability Assay (Promega Corp., Madison, WI) as described in Radio et al. (2008) [58]. Luminescence was measured thirty minutes after adding the reagent using a FLUOstar Optima plate reader (BMG LABTECH, Durham, NC).

## Results

### Microarray dose-response analyses

**Figure 1:**
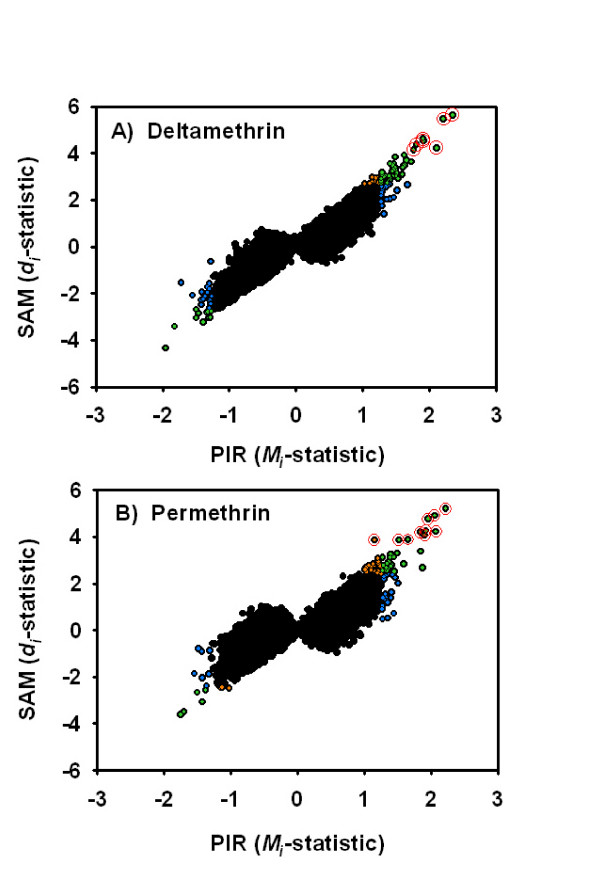
**Comparison of PIR and SAM regression methods**. Panels A & B plot the penalized isotonic regression (PIR) test statistic (*M*_*i*_, *x*-axis) against the penalized linear regression (SAM) test statistic (*d*_*i*_, *y*-axis) for deltamethrin and permethrin, respectively. All 31,042 probe sets present on the Affymetrix Rat 230 2.0 GeneChip^® ^are shown. Data points in green have an empirical *p*-value < 0.01 for both the PIR and SAM methods. Data points in blue have an empirical *p*-value < 0.01 for the PIR regression only. Data points in orange have an empirical *p*-value < 0.01 for the SAM regression only. In the deltamethrin and permethrin analyses, 49.5% and 53.7% of all probe sets identified by either the PIR or SAM method had *p *< 0.01 for both methods. Data points circled in red have a *q*-value < 0.10 in permutation-based *FDR *calculations employed in the SAM algorithm. Note that the rank order of statistical significance was similar between the two methods in that probe sets commonly identified using the PIR or SAM method tend to appear in the upper-right and lower left hand corners of the scatterplots (green points).

Both the PIR (isotonic) and SAM (linear) penalized regression methods identified dose-dependent increases and decreases in mRNA expression in the frontal cortex 6 h after an acute, oral exposure to both deltamethrin and permethrin. A comparison of the PIR and SAM regression models demonstrate that the two methods yield similar results in terms of identifying dose-responsive probe sets for both deltamethrin and permethrin (Figure 1A &1B). SAM analyses identified a small number of probe sets with dose-dependent increases in expression following either deltamethrin (n = 7) or permethrin (n = 10) exposure using the permutation-based FDR values as the significance criteria (*q *< 0.10, see Figure 1A &1B). The PIR analyses did not identify any probe sets for either pyrethroid with dose-dependent changes in expression at *q *< 0.10. A less statistically conservative method of identifying dose-related changes in probe set expression identified a larger number of significantly altered probe sets than that observed using the *FDR *criteria. Using a screening threshold of *p *< 0.01 the SAM analysis identified 70 and 61 probe sets with dose-dependent changes in expression for deltamethrin and permethrin, respectively, while the PIR analysis identified 93 and 85, respectively (Figure 1A–B). The overlap between probe sets identified as dose-responsive using the empirical *p*-value thresholds is considerable but incomplete. Overall, these parallel methods yield comparable results in that a rank-ordered list of dose-dependent changes in expression constructed using either the PIR or SAM test-statistics identifies the same groups of probe sets as being the most significantly changed within both the deltamethrin and permethrin test cohorts

To minimize the inclusion of false positives in qRT-PCR prioritization lists, all the probe sets for each compound that had empirical *p*-values < 0.01 in either the SAM or PIR regression methods were additionally analyzed with a one-way ANOVA with dose as the independent factor, followed by a Benjamini-Hochberg multiple testing correction (significance threshold, *p *< 0.05). For deltamethrin and permethrin, 95 of 109 (87.1%) and 53 of 89 (59.5%) probe sets passed the ANOVA significance threshold. The full list of probe sets considered significantly dose-responsive for deltamethrin (n = 95) and permethrin (n = 53) are listed in Additional files 3 and 4. Probe sets included in Additional files 3 and 4 that correspond to known protein-coding RefSeq database entries were considered candidates for qRT-PCR confirmation in dose-response Cohorts 3 and 4 and are listed in Tables 2 and 3.

**Figure 2:**
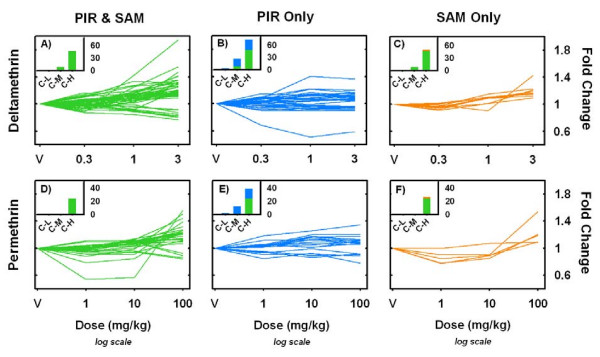
**Dose-response functions identified by PIR and SAM regression methods**. Panels A-F plot dose-response functions for probe sets identified by PIR (B & E), SAM (C & F) or both regression methods (A & D) for deltamethrin (A-C) and permethrin (D-F). Only probe sets that had a Benjamini-Hochberg adjusted *p*-value < 0.05 for a main effect of dose in a one-way ANOVA are shown. For each probe set expression summaries for each treatment group were normalized to vehicle control and plotted as fold-change from control. The color scheme corresponds to that used in Figure 1, with green curves being detected by both PIR and SAM regression methods (A, D), blue curves being detected exclusively with the PIR method (B, E) and orange curves being detected exclusively with the SAM method (C, F). Insets on each panel are the summated results of a Dunnett's many-to-one mean contrast test performed within each probe set comparing the means of the lowest (C-L), middle (C-M) and highest (C-H) doses to the mean of vehicle treated control. *y*-axis is number of probe sets identified under each comparison at a significance level of *p *< 0.05. Note the green portion of the stacked bars in the insets are the same values in inset panels A-C and D-E, respectively.

The dose-dependent changes in mRNA expression identified with the above analyses are relatively small in magnitude, < 2-fold change from control, and have varying patterns of expression across dose (Figure 2). Post-hoc analysis (Dunnett's mean contrast test) of these dose-response functions indicate that significant alterations in mRNA expression occur at doses below those needed to produce acute behavioral effects (Figure 2, insets). A majority of the probe sets identified as dose-responsive had mean expression values in the 3 mg/kg deltamethrin and 100 mg/kg permethrin dose groups different from those in the vehicle treated control group (78.9% and 77.3%, respectively). Of those probe sets, 25.3% and 19.5% also had mean expression values in the 1 mg/kg deltamethrin and 10 mg/kg permethrin dose groups different from controls. These latter doses are below those needed to produce acute neurotoxic effects on behavior. In addition, these data demonstrate that the PIR analyses detected a greater number of probe sets with mean expression values in the behavioral "NOAEL" dose groups (see Table 1) as being different from control as compared to the SAM analyses (compare Figure 2B &2E, insets to Figure 2C &2F, insets).

### Comparison of transcriptional effects across compounds

**Figure 3:**
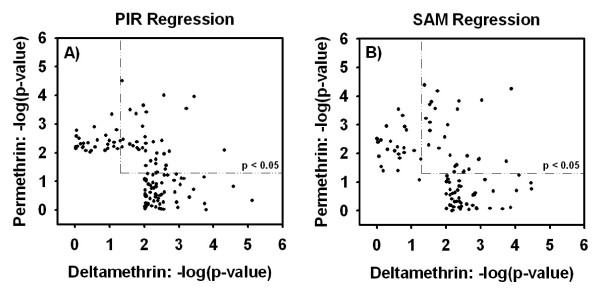
**Comparison of probe sets identified by PIR or SAM between pyrethroids**. Panels A and B plot the -log_10 _(empirical *p*-value) for deltamethrin (*x*-axis) against the -log_10 _(empirical *p*-value) for permethrin (*y*-axis) for probe sets identified during PIR or SAM regression analyses, respectively. All probe sets that had a Benjamini-Hochberg adjusted *p*-value < 0.05 in a one-way ANOVA for either permethrin or deltamethrin are included in the plot. Dashed boxes represent empirical *p*-value thresholds of *p *< 0.05. All points in the upper right of the figures, within the dashed boxes, meet the respective *p*-value criteria for both pyrethroids. 27.2% and 27.8% of all probe sets identified during PIR or SAM analysis, respectively, had empirical *p*-values of *p *< 0.05 for both compounds.

A comparison of the probe sets identified as dose-responsive in the PIR and SAM regression analyses demonstrates that the transcriptional response elicited by the two pyrethroids has some common characteristics. The panels in Figures 3 plot the -log_10 _of the empirical *p*-values associated with the PIR regression (3A) or SAM regression (3B) for each probe set identified as dose-responsive for either deltamethrin or permethrin. Data from the PIR regression analyses demonstrate that expression of 27.2% of all probe sets identified as dose-responsive for either pyrethroid are significantly altered by both compounds at an empirical *p*-value threshold of *p *< 0.05 (Figure 3A). Likewise, SAM analyses demonstrated that 27.8% of all dose-responsive transcripts are altered by both pyrethroids (Figure 3B). Differences in the global transcriptional response profiles between pyrethroids are also apparent.

### Quantitative real-time RT-PCR

Table 4 summarizes the results of the qRT-PCR assays and compares them to the fold-change expression values derived from the microarray study. Of the nine transcripts examined by qRT-PCR in Cohort 3, Ca^+2^/calmodulin dependent protein kinase 1γ (*Camk1g*) and dopa decarboxylase (*Ddc*) were commonly affected by both compounds indicating that for these genes there was no differences in the changes in expression elicited by equipotent doses of either pyrethroid. *Camk1g *qRT-PCR expression values closely resembled those observed in the microarray study. In contrast to *Camk1g*, the microarray dose-response cohort demonstrated a dose-related change in *Ddc *expression for deltamethrin only, even though a clear dose-dependent decrease in *Ddc *mRNA expression was observed in both the deltamethrin and permethrin qRT-PCR cohorts.

A significant interaction between compound and EDL was observed for glycerol-3-phosphate dehydrogenase 1 (*Gpd1*) and FK506-binding protein 5 (*Fkbp51*), indicating that equipotent doses of the two pyrethroids did not elicit similar changes in expression at 6 h post-exposure. A main effect of dose was observed for *Gpd1 *and *Fkbp51 *mRNA only for deltamethrin (Table 4). The qRT-PCR expression values for *Gpd1 *and *Fkbp51 *closely match those observed in the microarray study.

The immediate early genes (IEG), FBJ murine osteosarcoma viral oncogene homolog (*c-fos*) and early growth response 1 (*Egr1*) were differentially affected by the two pyrethroids at 6 h post-exposure, however, no significant main effect of dose (EDL) was observed for either compound. For deltamethrin, the direction of fold-change for *c-fos *and *Egr1 *is down in most dose groups measured by qRT-PCR. In contrast, for permethrin no change in the expression of *c-fos *and *Egr1 *mRNA was observed across dose groups in the qRT-PCR cohort. While *c-fos *and *Egr1 *expression at 3 mg/kg deltamethrin and 100 mg/kg permethrin reflect the direction of fold-change observed in the microarray study, very little similarity is apparent between qRT-PCR and microarray expression values for these genes at the lower dose levels (Table 4).

There were no effects of pyrethroid exposure on mRNA expression for heat shock 27 kDa protein (*Hsp27*), brain derived neurotrophic factor (*BDNF*) or Ras association (RalGAS/AF-6) domain family 6 (*Rassf5*) (data not shown). In the case of *BDNF*, qRT-PCR expression values closely approximate the expression values observed in a second probe set not identified as dose-responsive in the microarray analyses (data not shown).

**Figure 4:**
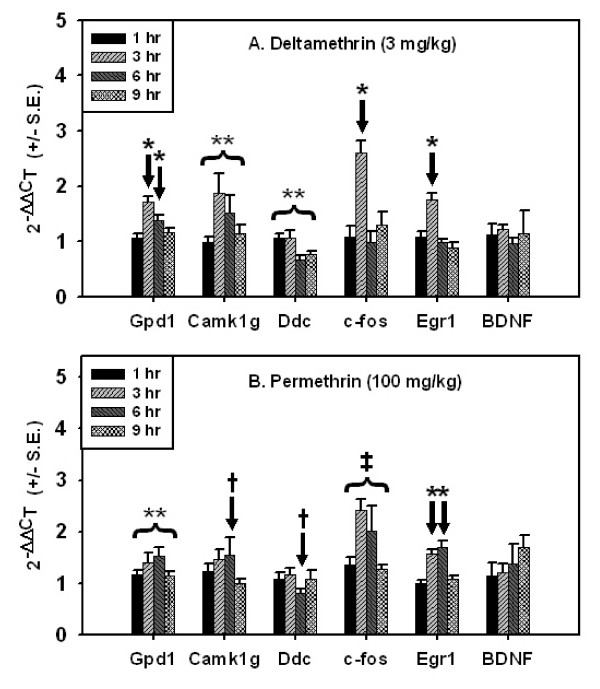
**qRT-PCR time course results**. Transcript expression over time following a single acute dose of 3 mg/kg deltamethrin (top) or 100 mg/kg permethrin (bottom). Gene symbols are listed on the *y*-axis. Data were analyzed using two-way ANOVA followed by one-way ANOVA within time points were interaction was observed. (**) denotes no interaction of time and treatment and a main effect of treatment (*p *< 0.05). (‡) denotes no interaction of time and treatment and a main effect of both time and treatment (*p *< 0.05). (*) denotes a significant effect of treatment for that time point (*p *< 0.05). (†) denotes a significant main effect of dose from qRT-PCR dose-response analysis (Table 4). Values for time-matched vehicle controls are not shown. A summary of the statistical analyses performed on these data is provided in Additional file 5.

Characterization of the time course of mRNA expression for *Camk1g*, *Gpd1*, *c-fos *and *Egr1 *demonstrates that altered expression of these transcripts also occurs at times earlier than 6 h following acute, oral pyrethroid exposure (Figure 4, Additional file 5). Treatment-related increases in *Camk1g *and *Gpd1 *mRNA expression were observed for both deltamethrin and permethrin. For deltamethrin, both *Camk1g *and *Gpd1 *mRNA had maximally induced expression at 3 h followed by persistent elevations at 6 h (Figure 4). For permethrin, both *Camk1g *and *Gpd1 *had maximal induction at 6 h preceded by slight elevations at 3 h. Permethrin-mediated *Gpd1 *induction was statistically significant while *Camk1g *induction reflected the trends observed in the dose-response cohorts but did not reach statistical significance. *Ddc *mRNA expression was decreased following both deltamethrin and permethrin exposure. For deltamethrin decreased expression began at 6 h and persisted through 9 h while for permethrin, expression decreased at 6 h only. The changes in *Ddc *mRNA expression over time were consistent with those observed in the qRT-PCR cohort

Both deltamethrin and permethrin increase the expression of the IEGs *c-fos *and *Egr1*, albeit with different temporal characteristics. Expression of *c-fos *and *Egr1 *increases at 3 h for deltamethrin and returns to control levels at 6 h. For permethrin, expression of *c-fos *and *Egr1 *increases at 3 h, remains persistently elevated at 6 h and returns to control levels by 9 h. The large increases in *c-fos *and *Egr1 *for permethrin and not deltamethrin are consistent with the microarray data as these two genes were identified as dose-responsive at 6 h for the former and not the latter compound. However, the data in Figure 4 demonstrate that the two pyrethroids, in fact, elicit qualitatively similar responses in the expression of *c-fos *and *Egr1*. The expression of another IEG, *BDNF*, is apparently not affected by pyrethroids under the dosing paradigm used in this study.

### Significant Analysis of Function and Expression (SAFE)

Seven GO categories were identified as commonly enriched for both pyrethroids using SAFE analysis and Fisher's χ^2 ^method (Table 5). The composition of the commonly enriched categories for both chemicals included genes involved in neuronal morphogenesis, intracellular Ca^+2 ^signaling and small molecule transport. In addition, five GO-BP categories and two canonical KEGG pathways were identified as enriched in the individual SAFE analyses of permethrin and deltamethrin, respectively (Table 5). For permethrin, the SAFE findings include enriched gene categories related to neuronal morphogenesis and developmental patterning. For deltamethrin the SAFE findings include two KEGG metabolic pathways, one of which involves synthesis of the precursor molecules for monoamine neurotransmitters.

**Figure 5:**
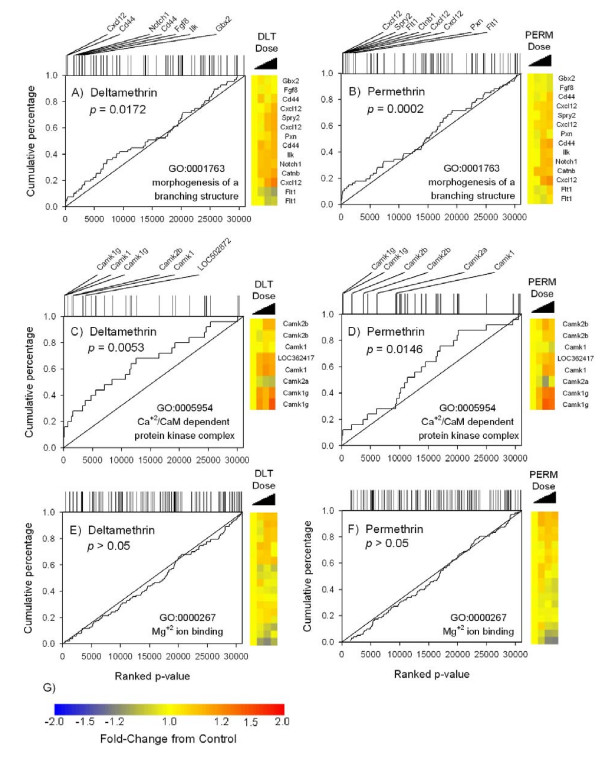
**Composition and expression patterns of significantly enriched GO categories from SAFE analysis**. Panels A-D are SAFE plots for two commonly enriched categories for both deltamethrin (A & C) and permethrin (B & D). Panels E & F are SAFE plots for a category not enriched for either deltamethrin (E) or permethrin (F). The *x*-axis of each plot denotes the position of all probe sets in a rank ordered list of significance (from left to right) according to the empirical *p*-value from a linear regression across dose. The *y*-axis is a cumulative percentage calculated by taking the rank position of a given probe set either within the entire data set (solid unity line) or the interrogated Gene Ontology sub-category (solid stair-step line) and dividing them by the total number of probe sets contained within the entire data set or interrogated category, respectively. The degree of deviation of the stair-step line from the unity line indicates enrichment. The probe sets (excluding ESTs) that are ranked highest in significance for each GO category for both compounds in panels A-B and C-D are denoted at the top of each panel and included in a heatmap to the side of the respective panels. In the heatmaps, each row of tiles is a probe set and each column of tiles represents the mean fold-change from control with increasing doses of each compound running from right to left. Colorbar for heatmaps is given in panel G.

SAFE plots of the GO categories 'morphogenesis of a branching structure' and 'Ca^+2^/calmodulin dependent protein kinase complex' demonstrate the significant category enrichment for both permethrin and deltamethrin (Figure 5). This is evidenced by the divergence of the stair step line from the unity line near the far left of Figure 5, panels A-D. A SAFE plot of a GO category not significantly enriched for either compound is given in Figure 5, panels E-F for comparison purposes. The most significant dose-responsive transcripts for each of the enriched GO categories are illustrated in the heatmaps to the right of Figure 5, panels A-D. These heatmaps demonstrate that appreciable dose-dependent increases in the expression of probe sets contained within the enriched GO categories occurs following pyrethroid exposure.

### Pyrethroid effects on neurite length and branching in primary mixed cortical cell cultures

**Figure 6:**
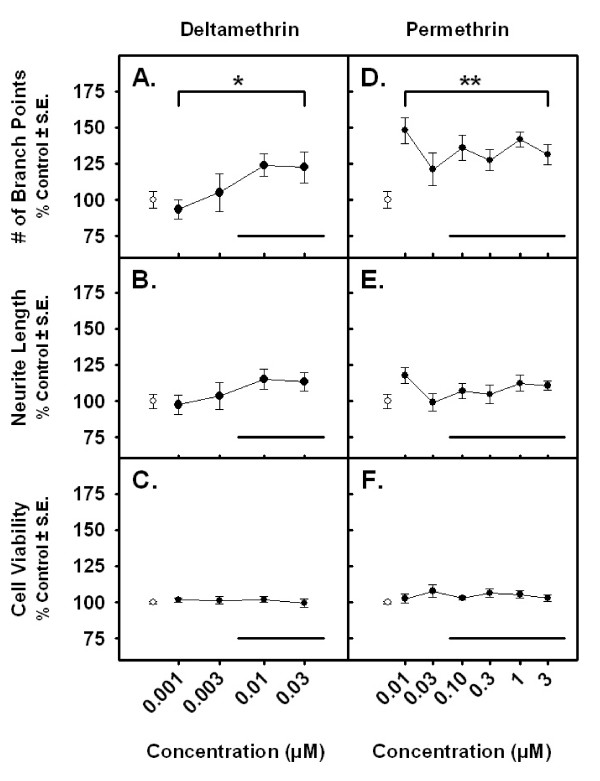
**Pyrethroid effects on branching and neurite length in primary cortical cell cultures**. Changes in the total number neurite of branch points (A & D), total neurite length (B & E) and cell viability (C & F) in primary cortical cell cultures exposed to deltamethrin (A-C) or permethrin (D-F). n = 3 replicate experiments. Values for each end point are normalized to untreated controls (± standard error). Untreated control values are shown in white. The bold lines underneath each curve represent the range of estimated brain concentrations expected to occur during the *in vivo *exposures used in the present study (Mirfazaelian et al. 2006 and Tornero-Velez et al. 2007). Significance was determined using a One-way ANOVA, * = *p *< 0.10, ** = *p *< 0.05.

Both deltamethrin and permethrin produce an increase in the number of neurite branch points following a 96 h exposure (Figure 6A &6D). The range of predicted tissue concentrations (in μM) from the pharmacokinetic predictions listed in Table 6 are marked near the *x*-axes and correspond well to areas along the *in vitro *dose-response curve where changes in branching were observed. An average increase of ~25% above control in the number of neurite branch points was observed at nominal media concentrations ranging from 0.01 – 0.03 μM deltamethrin and 0.01 – 3 μM permethrin. No significant increase in total neurite length was observed for either compound save at the 0.01 μM exposure level for permethrin (Figure 6B &6E). Changes in cell viability were not apparent in the concentration ranges tested (Figure 6C &6F).

## Discussion

A principle finding of the present study was that dose-dependent alterations in gene transcription occur in the cortex at doses of deltamethrin and permethrin below those required to elicit acute neurotoxic effects in the whole animal. Both similarities and differences in the overall transcriptional response were observed when comparing the two pyrethroids. Quantitative real-time RT-PCR analysis in additional cohorts of animals provided independent biological and technical replicates of the findings from the microarray data set. In addition, transcripts for which the time course of gene expression was characterized demonstrated qualitative similarities in the response for both pyrethroids. SAFE analysis of the microarray data identified several GO categories jointly enriched by both deltamethrin and permethrin including some related to branching morphogenesis. Subsequently, a significant increase in the number of neurite branch points was observed in a primary cortical cell culture model.

### Microarray dose-response analyses

Dose-dependent alterations in transcript expression were observed in frontal cortex 6 h following acute exposure to pyrethroids. Prior to experimentation, the shape of the dose-response curve for any potential alterations in gene transcription was unknown. Visual inspection of the data demonstrated a definite heterogeneity in the types of dose-response functions produced by these pyrethroid exposures (see Figure 2). The biological factors mediating this heterogeneity are unclear but may involve activation of different intracellular signaling pathways at different points along the dose range [59-62]. To generate lists of candidate genes for qRT-PCR follow-up that captures this heterogeneity, the GeneChip^® ^expression data was analyzed using two independent but similarly structured regression methods: SAM and a novel PIR. Both methods identified qualitatively similar dose-related alterations in gene expression within each compound (Figure 1A &1B). The SAM regression model detected a number of dose-responsive transcripts with expression levels different from control only at the highest pyrethroid dose (Figure 2). However, PIR identified other dose-related changes with small, but significant, increases or decreases in expression that were similar in magnitude at both the "NOAEL" and "high" pyrethroid doses (Figure 2B &2E) and not detected by SAM. These dose-responsive transcripts may in fact be biologically relevant responses to acute pyrethroid intoxication which would have been excluded using the standard SAM analysis. Therefore, while both SAM and PIR identified the same sub-set of transcripts within compound, the PIR method detected a larger component of the global transcriptional response of the cortex composed of expression changes that do not fit a linear model.

The regression analysis frameworks detailed in this work were used exclusively as an identification and prioritization method for selection of genes for subsequent qRT-PCR analyses. Conclusions concerning the biological significance of individual transcriptional changes were reserved for those transcripts successfully replicated by qRT-PCR in independent cohorts of test subjects. For the goals of this study, the risk of excluding true positives in the microarray data analysis outweighed the caveat of including false positives from the final list of prioritized targets. Thus, the modified protocol of regression screening and subsequent ANOVA based analyses was adopted.

### Comparison across compounds

The present data demonstrate both similarities and differences in the global transcriptional response in rat cortex to acute, low-dose deltamethrin and permethrin exposure. Similarities in the global transcriptional response across compounds suggest that these two pyrethroids may affect common biological pathways (Figure 3). The differences observed between compounds in the global transcriptional response (i.e. microarray dataset) are likely due to a combination of two factors: 1) authentic heterogeneity in the pharmacodynamic activities of deltamethrin and permethrin on gene transcription and 2) a slight offset in the time course of qualitatively similar responses across compounds. In addition, time course data implies that additional alterations in gene expression not detected in the 6 h dose-response study may occur at time points other than the one sampled. The qRT-PCR data shown here support this conclusion (Figure 4).

The results of the SAFE functional category level analysis support the conclusion that the biological activities of the two pyrethroids overlap. Several categories were found to be commonly upregulated between the two compounds. Similarities are not surprising, given that both pyrethroids act on mammalian VSSCs [12,32]. Whether the individual gene changes or impacted functional categories are directly linked to this site of action, remains yet to be determined. Importantly, these data provide guidance on some novel cellular functions affected by pyrethroids.

### Biological significance of experimental findings

Interestingly, probe sets corresponding to the primary molecular targets for pyrethroids were not altered for either pyrethroid tested in the microarray study. Specifically, there were no treatment related changes in any of the VSSC or VSCC isoforms/subunits or any subunits that comprise neurotransmitter receptors complexes [63-66]. This finding is supported by *in vitro *data [27] that characterized the global transcriptional response of cortical neurons exposed to a variety of pharmacological agents that altered firing rates. No changes in the expression of VSSC or VSCC isoforms/sub-units or neurotransmitter receptors were identified in this study in response to increases in neuronal firing rates [27]. Since a primary action of pyrethroids is to change firing rates [67], the present data do not support transcriptional induction or repression of VSSCs, VSCCs or neurotransmitter receptor subunits as a neuronal response to acute pyrethroid exposure. These data do not exclude transcription-independent changes in the expression or functional state of these channels known to occur following excitatory stimuli [68-71].

The immediate early transcription factors *c-fos *and *Egr1 *were upregulated by deltamethrin and permethrin. This is consistent with IEG expression changes in the cortex following acute pyrethroid exposure [29,72]. Increased *Egr1 *and *c-fos *expression supports that deltamethrin and permethrin increased neuronal excitation in the present study. *Egr1 *and *c-fos *are among the genes induced by increased neuronal firing in cortical cells in culture [27], as well as *in vivo *following stimuli that produce neuronal excitation [73,74]. Induction of IEG mRNAs is a rapid transcriptional response of neurons following increased activity [75-78]. The time course for the expression of the IEGs *c-fos *and *Egr1 *does not support *de novo *gene transcription as being responsible for mediating the acute behavioral effects of pyrethroids. The earliest time that increased IEG expression is observed in the present study is at 3 h: IEG expression is at control levels at 1 h. Onset of behavioral effects following oral pyrethroid exposure occurs prior to the onset of increased IEG expression (i.e. 30 min – 1 h) [79]. Therefore, the IEG induction described here can not mediate the acute neurotoxic signs of pyrethroid intoxication, but instead are markers of neuronal excitation.

The present study found dose- and time-dependent increases in the expression of *Camk1g *mRNA. Data from *in vitro *models of developmental morphogenesis in neurons indicates that increased expression of *Camk1g *(Table 4 and Figure 4) may alter the structure and function of pyrethroid-sensitive neurons. Wayman et al. (2006) [80] demonstrated that *Camk1g *plays a specific role in the activity-dependent growth of hippocampal neurons between 7-9DIV by activating a Ras/MEK/ERK/CREB/*Wnt2 *signaling cascade in response to excitatory stimuli. In addition, Takemoto-Kimura et al. (2007) [81] demonstrated that *Camk1g *participates in a Rac signaling pathway that mediates the morphogenesis of cortical neurons. In both those studies, artificial knockdown or over-expression of *Camk1g *altered outgrowth of neuronal processes in a development context [80,81]. The role of *Camk1g *in maintenance and plasticity of neuronal processes in the adult CNS is currently unknown. Furthermore, there is evidence in the literature that *Camk1g *mRNA expression is regulated by changes in neuronal firing patterns similar to IEGs. Changes in neuronal firing rates correlates with increases or decreases in the expression of *Camk1g *mRNA [27,82-85]. These observations support that pyrethroid-mediated changes in neuronal firing rates could mediate changes in the expression of *Camk1g*, which may in turn lead to changes in neuronal morphology (see below), especially during development.

The transcriptional upregulation of glycerol-3-phosphate dehydrogenase 1 (*Gpd1*) and FK506-binding protein (*Fkbp51*) mRNA (Table 4, Figure 4) indicate: 1) that pyrethroid exposure activates the hypothalamic-pituitary-adrenal (HPA) axis and 2) that non-neuronal cell populations in the CNS are sensitive to pyrethroids. The proteins encoded by *Gpd1 *and *Fkbp51 *are expressed in the brain exclusively in oligodendrocytes [86] and T-cell lymphocytes [87]. Both the *Gpd1 *and *Fkbp51 *genes contain glucocorticoid receptor binding motifs either in the upstream promoter region (*Gpd1*) [88] or in an intronic region (*Fkbp51*) [89] and increased expression of both is dependent upon glucocorticoid hormone stimulation [90,91]. Glucocorticoids are released in the circulation from the adrenals in response to a variety of stressors and increased circulating corticosterone levels were reported in the rat following deltamethrin exposures; albeit at very high, intravenous doses [92]. It is likely that increases in *Gpd1 *and *Fkbp51 *expression may be components of a generalized, non-specific stress response brought about by overstimulation of the HPA axis by pyrethroids. The potential impact of increased *Gpd1 *and *Fkbp51 *expression on the health and function of affected glia, to date, is unclear.

Decreases in the expression of aromatic L-amino acid decarboxylase (*Ddc*) suggest that pathways controlling monoaminergic neurotransmitter synthesis may be affected by pyrethroids. *Ddc *is the final enzyme in the synthesis pathways of dopamine and serotonin [93]. Previous reports note a depletion of dopamine and serotonin in a variety of brain regions following repeated exposure to deltamethrin [94-96]. In the case of dopamine depletion, two of these studies demonstrate concurrent decreases in the expression of tyrosine hydroxylase, the penultimate enzyme in dopamine synthesis [95,96]. The mechanism controlling *Ddc *mRNA repression following pyrethroid exposure is unclear, but provides support that monoaminergic neurotransmitter systems are sensitive to the compounds.

The changes in gene transcription observed in the present study occur at doses at or near the threshold for eliciting acute neurobehavioral signs of intoxication in the whole animal [33,97,98]. Time course data (Figure 4) demonstrate that transcriptional changes are transient and consistent with the onset and recovery of actue behavioral effects following acute, oral exposures observed in previous studies [97,99]. Currently, it is unclear if these transient gene expression changes are simply an adaptive response of the nervous system to excitation by pyrethroids, or whether they may contribute to development of an adverse health effect. Regardless, these data demonstrate alterations in gene transcription in cortex at low doses of pyrethroids that produce only mild effects observed in the whole animal.

Overall, increased expression of *c-fos*, *Egr1 *and *Camk1g *in the present study are most likely regulated by pyrethroid-induced changes in the neuronal firing patterns of cortical neurons. The increased expression of *Gpd1 *and *Fkbp51 *mRNA indicates an indirect effect on glia due to non-specific activation of the HPA-axis.

### Pyrethroid effects on branching morphogenesis

The SAFE analyses yielded an enrichment of the category 'morphogenesis of a branching structure' for both pyrethroids. This was due to dose-dependent changes in expression at 6 h for several genes that control neurite branching and morphogenesis including *Cxcl12, Notch1 *and β-catenin [100-102]. A major function of this group of genes is thought to involve the regulation of neuronal morphogenesis during development. It is unknown whether the same gene categories would show enrichment at sampling times other than 6 h or whether these transient changes in gene expression leads to a significant change in neuronal morphology in the adult cortex. Herein, we also report that both pyrethroids increased neurite branch points in a developmental model of neurite growth [58], but did not alter total neurite length (Figure 6). The gene expression data from the present study are consistent with pyrethroid effects on neurite branching and not neurite length.

Overexpression of *Notch1 *in rat cortical neurons results in an increase in neuronal branching and an antagonism of neurite extension [100]. Likewise, overexpression of β-catenin and *Cxcl12 *results in increased dendritic and axonal branch tip number, respectively, and has no or opposite effects on measures of length [101,102]. Transcripts for these genes are upregulated following pyrethroid exposure in the present microarray data (see Figure 5 heatmaps) and suggest that pyrethroids affect the developmental morphogenesis of neurons. However, these data are not consistent with the results of two previous studies of pyrethroid effects on developmental neuronal outgrowth. Treatment of developing *X. laevis *neurons with 10 nM deltamethrin resulted in an increase in total neurite length in the presence of extracellular Ca^+2^[103]. In contrast, exposure of N2A neuroblastoma or C6 glioma cells to the pyrethroid cypermethrin resulted in no effect on morphology [104]. The disparity between the results from these studies and the present study may be due to differences in the experimental conditions (cell types, media, exposure durations, etc.), all of which are known to impact neuronal outgrowth [105]. Preliminary experiments in PC-12 neuroblastoma cells (*data not shown*) did not demonstrate any effects on neurite branching or length. The present data is the first to demonstrate an effect of pyrethroids on the branching morphology of primary cultured neurons.

Disruption of neuronal morphogenesis in the developing nervous system by pyrethroids could result in detrimental effects on neurological function later in life. Intermittent exposure to stimulant drugs such as amphetamine can produce an increase in dendritic branching *in vivo *in both juvenile and adult rats [106-108]. These morphological changes are hypothesized to underlie some of the adverse neurological effects associated with abuse of stimulant drugs (e.g., learning deficits) [106,109]. In addition, lead exposure during development results in neurological deficits that have been associated with changes in neuronal morphology [110-112]. Both lead and stimulant drugs facilitate neurite outgrowth in *in vitro *cell culture models that is similar, but not identical, to the increased branching observed with pyrethroids in the present study [113-117]. Several questions remain to be addressed before definitive conclusions regarding pyrethroid effects on neuronal morphogenesis can be made, including: 1) whether or not pyrethroid-induced changes in morphology occur *in vivo*, 2) are effects on morphogenesis specific to cortical neurons, and 3) do all compounds in the pyrethroid class produce the same types of effects on neuronal branching morphogenesis?

## Conclusion

The present study has identified a group of genes whose transcription is altered in a dose-dependent manner in the rat cortex following *in vivo *pyrethroid exposure. A majority of the gene expression changes observed in this study are consistent with the induction of neuronal hyperexcitability by pyrethroids. The gene expression changes observed are transient, comparable between the two pyrethroids tested and provide insight into the cellular response of the neurons downstream of the pharmacological effects of these compounds at the neuronal membrane. Most importantly, this study provides evidence that branching of cortical neurons is increased by pyrethroids, suggesting the neurotoxic action of these compounds may include effects on neuronal morphology.

## Abbreviations

*B3galt3*: UDP-Gal:betaGlcNAc beta 1,3-galactosyltransferase, polypeptide 3; *Bcat*: branched chain aminotransferase 1, cytosolic; *Bdnf*: brain derived neurotrophic factor; *Bves*: blood vessel epicardial substance; *c-fos*: FBJ murine osteosarcoma viral oncogene homolog; *Camk1g*: calcium/calmodulin-dependent protein kinase I gamma; *Crh*: corticotropin releasing hormone; *Cryab*: crystallin, alpha B; *Cxcl12*: chemokine (C-X-C motif) ligand 12; *Ddc*: dopa decarboxylase; *Dusp6*: dual specificity phosphatase 6; *Dync1i1*: dynein cytoplasmic 1 intermediate chain 1; *EDL*: equipotent dose level; *Egr1*: early growth response 1; *Ets2*: v-ets erythroblastosis virus E26 oncogene homolog 2; *Fbxo22*: F-box only protein 22; *Finb*: ras responsive element binding protein 1 (predicted); *Fkbp51*: FK506 binding protein 5; GCOS: GeneChip^® ^Operating Software; *Gna14*: guanine nucleotide binding protein, alpha 14; *Gpd1*: glycerol-3-phosphate dehydrogenase 1; *Heatr1*: HEAT repeat containing 1 (predicted); HPA – hypothalamic-pituitary-adrenal axis; *Hsp27*: heat shock 27 kDa protein 1; *Hyou1*: hypoxia up-regulated 1; *Igfpb3*: insulin-like growth factor binding protein 3; *Klf4*: Kruppel-like factor 4; *Klf10*: Kruppel-like factor 10; *Lrg1*: leucine-rich alpha-2-glycoprotein 1; *Lpen2*: lipin 2 (predicted); *Max*: Max protein; *Medl19*: mediator of RNA polymerase II transcription, subunit 19 homolog; mRNA: messenger ribonucleic acid; *Nedd4l*: neural precursor cell expressed, developmentally down-regulated 4-like; *Nr4a3*: nuclear receptor subfamily 4, group A, member 3; *Pde10a*: phosphodiesterase 10A; *Pdlm7*: PDZ and LIM domain protein 7; PIR: penalized isotonic regression; *Pld1 *- phospholipase D1; *Polr2c*: polymerase (RNA) II (DNA directed) polypeptide C, 33 kDa; *Prim2*: DNA primase, p58 subunit; qRT-PCR: quantitative real-time polymerase chain reaction; *Rassf5*: ras association (RalGDS/AF-6) domain family 5; *Ret*: ret proto-oncogene; *Rimbp2*: RIMS binding protein 2; *Rkhd3*: ring finger and KH domain containing 3 (predicted); RMA: Robust Multi-array Average; SAFE: Significant Analysis of Function and Expression; SAM: Significant Analysis of Microarrays; *Siat7E*: sialyltransferase 7E; *Slc39a8*: solute carrier family 39 (zinc transporter), member 8; *Slc40a1*: solute carrier family 40 (iron-regulated transporter), member 1; *Slit2*: slit homolog 2; *Sta2*: stefin A2 (predicted); *Tcfcp2l1*: transcription factor CP2-like 1; *Timp3*: tissue inhibitor of metalloproteinase 3; *Tmem10*: transmembrane protein 10; *Usp54*: ubiquitin specific peptidase 54; *Vdac1*: voltage-dependent anion channel 1; *Wrnip1*: Werner helicase interacting protein 1; *Xdh*: xanthine dehydrogenase; *Zcch8*: zinc finger, CCHC domain containing 8 (predicted)

## Authors' contributions

JAH guided the study designs, carried out all RNA extractions, qRT-PCR experiements and data analyses, performed microarray data analyses and drafted the manuscript. FAW and ZL performed the penalized isotonic regression and SAFE analyses and provided valuable input on microarray analysis methods. NMR and WRM performed neuronal morphogenesis assays. RTV provided pharmacokinetic predictions of target tissue doses. KMC participated in study design, manuscript preparation and interpretation of this work.

## Supplementary Material

Additional file 1 ***Comparison of mean coefficients of variation (CV) between GCOSv1.2 and RMA microarray expression summaries***. For each expression summary calculation method, all 31,042 probe sets present on the Affymetrix Rat 230 2.0 GeneChip^® ^array were sorted based on the mean expression summary within the control group and divided into equally sized percentile ranges in ascending order. CV's were calculated for each individual probe set within each dose group. The mean CV for each percentile range was then calculated across probe sets for each dose group. Expression summaries calculated using RMA consistently reduces the variability of the expression summaries across the entire data set when compared to GCOSv1.2. A dramatic decrease in variability is observed in the lower 50% of the data set. Click here for file

Additional file 2 **Taqman^® ^qRT-PCR assay information**. Click here for file

Additional file 3 ***List of probe sets with dose-dependent changes in expression for deltamethrin***. Affymetrix probe set IDs without a gene symbol are expressed sequence tags (ESTs). Probe sets with arrows correspond to genes examined by qRT-PCR. Positive SAM *d*_*i *_or PIR *M*_*i *_scores denote upregulated probe sets. Negative SAM *d*_*i *_or PIR *M*_*i *_scores denote downregulated probe sets. Click here for file

Additional file 4 ***List of probe sets with dose-dependent changes in expression for permethrin***. Affymetrix probe set IDs without a gene symbol are expressed sequence tags (ESTs). Probe sets with arrows correspond to genes examined by qRT-PCR. Positive SAM *d*_*i *_or PIR *M*_*i *_scores denote upregulated probe sets. Negative SAM *d*_*i *_or PIR *M*_*i *_scores denote downregulated probe sets. Click here for file

Additional file 5 **Two-way analysis of variance (ANOVA) for qRT-PCR time course data**. Click here for file
