# Supplementary material for: Transcriptional response of rat frontal cortex following acute In Vivo exposure to the pyrethroid insecticides permethrin and deltamethrin
Source: BMC Genomics. 2008 Nov 18;9:546. doi: 10.1186/1471-2164-9-546 (PMC2626604; doi:10.1186/1471-2164-9-546)
Supplement: Additional file 1 — Comparison of mean coefficients of variation (CV) between GCOSv1.2 and RMA microarray expression summaries. For each expression summary calculation method, all 31,042 probe sets present on the Affymetrix Rat 230 2.0 GeneChip® array were sorted based on the mean expression summary within the control group and divided into equally sized percentile ranges in ascending order. CV's were calculated for each individual probe set within each dose group. The mean CV for each percentile range was then calculated across probe sets for each dose group. Expression summaries calculated using RMA consistently reduces the variability of the expression summaries across the entire data set when compared to GCOSv1.2. A dramatic decrease in variability is observed in the lower 50% of the data set. [file 1471-2164-9-546-S1.doc]

| **Mean coefficients of variation for GCOSv1.2 normalized expression summaries** | | | | | | | | | | |
| --- | --- | --- | --- | --- | --- | --- | --- | --- | --- | --- |
|  | | | | | | | | | | |
|  | **1-10%** | **11-20%** | **21-30%** | **31-40%** | **41-50%** | **51-60%** | **61-70%** | **71-80%** | **81-90%** | **91-100%** |
|  |  |  |  |  |  |  |  |  |  |  |
| **n** | 3105 | 3104 | 3104 | 3104 | 3104 | 3104 | 3104 | 3104 | 3104 | 3105 |
|  |  |  |  |  |  |  |  |  |  |  |
| **Control** | 66.52 | 57.09 | 40.99 | 32.80 | 28.50 | 26.69 | 25.29 | 24.84 | 24.33 | 21.21 |
| **Permethrin** |  |  |  |  |  |  |  |  |  |  |
| **1 mg/kg** | 63.15 | 55.64 | 41.30 | 33.61 | 29.65 | 29.05 | 28.67 | 28.48 | 28.24 | 24.44 |
| **10 mg/kg** | 63.61 | 55.24 | 40.35 | 32.25 | 27.69 | 25.73 | 25.08 | 24.45 | 23.72 | 21.13 |
| **100 mg/kg** | 61.74 | 55.51 | 41.06 | 33.35 | 28.94 | 27.03 | 26.02 | 25.35 | 24.78 | 22.55 |
| **Deltamethrin** |  |  |  |  |  |  |  |  |  |  |
| **0.3 mg/kg** | 61.29 | 52.91 | 37.26 | 28.68 | 24.00 | 21.44 | 19.17 | 17.85 | 16.91 | 14.91 |
| **1.0 mg/kg** | 61.36 | 54.54 | 39.81 | 31.28 | 27.01 | 24.69 | 23.65 | 23.00 | 22.55 | 19.61 |
| **3.0 mg/kg** | 63.26 | 55.61 | 39.18 | 32.80 | 25.77 | 23.49 | 21.79 | 20.53 | 19.52 | 17.03 |
|  |  |  |  |  |  |  |  |  |  |  |
| **Mean coefficients of variation for RMA normalized expression summaries** | | | | | | | | | | |
|  | | | | | | | | | | |
|  | **1-10%** | **11-20%** | **21-30%** | **31-40%** | **41-50%** | **51-60%** | **61-70%** | **71-80%** | **81-90%** | **91-100%** |
| **n** | 3105 | 3104 | 3104 | 3104 | 3104 | 3104 | 3104 | 3104 | 3104 | 3105 |
|  |  |  |  |  |  |  |  |  |  |  |
| **Control** | 7.08 | 9.99 | 11.95 | 13.65 | 15.05 | 16.59 | 18.07 | 18.45 | 19.21 | 16.86 |
| **Permethrin** |  |  |  |  |  |  |  |  |  |  |
| **1 mg/kg** | 7.39 | 10.53 | 12.77 | 15.25 | 17.36 | 20.03 | 22.71 | 23.35 | 23.80 | 20.53 |
| **10 mg/kg** | 6.99 | 9.68 | 11.66 | 13.69 | 15.40 | 17.47 | 19.42 | 20.21 | 20.72 | 18.08 |
| **100 mg/kg** | 7.01 | 10.10 | 12.27 | 14.41 | 16.52 | 18.68 | 20.99 | 21.76 | 22.43 | 19.46 |
| **Deltamethrin** |  |  |  |  |  |  |  |  |  |  |
| **0.3 mg/kg** | 6.08 | 8.40 | 9.96 | 11.42 | 12.55 | 13.45 | 14.33 | 14.51 | 14.89 | 12.84 |
| **1.0 mg/kg** | 6.66 | 9.49 | 11.49 | 13.34 | 14.95 | 16.71 | 18.55 | 19.16 | 19.75 | 17.70 |
| **3.0 mg/kg** | 6.53 | 8.99 | 10.84 | 13.65 | 13.94 | 15.37 | 16.58 | 17.09 | 17.27 | 15.29 |

**Additional File 1. *Comparison of mean coefficients of variation (CV) between GCOSv1.2 and RMA microarray expression summaries.*** For each expression summary calculation method, all 31,042 probe sets present on the Affymetrix Rat 230 2.0 GeneChip® array were sorted based on the mean expression summary within the control group and divided into equally sized percentile ranges in ascending order. CV’s were calculated for each individual probe set within each dose group. The mean CV for each percentile range was then calculated across probe sets for each dose group. Expression summaries calculated using RMA consistently reduces the variability of the expression summaries across the entire data set when compared to GCOSv1.2. A dramatic decrease in variability is observed in the lower 50% of the data set.
