# Supplementary material for: Transcriptional response of rat frontal cortex following acute In Vivo exposure to the pyrethroid insecticides permethrin and deltamethrin
Source: BMC Genomics. 2008 Nov 18;9:546. doi: 10.1186/1471-2164-9-546 (PMC2626604; doi:10.1186/1471-2164-9-546)
Supplement: Additional file 2 — Taqman® qRT-PCR assay information. [file 1471-2164-9-546-S2.doc]

## **Additional File 2. *Taqman® qRT-PCR assay information****.*

Gene Amplicon Amplification

Symbol Assay I.D. Reference Sequence Assay Position* Length Efficiency (%)

β-actin Rn00667869_m1 CTTCCTTCCTGGGTATGGAATCCTG 4 - 5 exon junction 91 95.2

Gpd1 Rn00573596_m1 GGGCCTCGTGGACAAGTTTCCCTTG 7 - 8 exon junction 79 92.4

Fkpb51 Rn01768371_m1 GAGCAGGATGCCAAGGAAGAGGCCA 10 - 11 exon junction 74 91.8

Camk1g Rn00788224_m1 CATTTCTGAGTCAGCCAAGGACTTT 8 - 9 exon junction 71 96.0

Hsp27 Rn00583001_g1 TCACCCGGAAATACACGCTCCCTCC 2 - 3 exon junction 136 94.4

Ddc Rn01401187_m1 TCCGGCTAAAGGGCTCCAACCAGTT 13 - 14 exon junction 102 92.5

BDNF Rn02531967_s1 AAATTCTTGCTGTGGTCTCTTTTTG exon VIII** 142 97.5

Rassf5 Rn00571287_m1 GGAGACGTAGAGTGGGATGCCTTTT 5 - 6 exon junction 75 92.4

c-fos Rn02105433_s1 CTTCAGCGTCCATGTTCATTGTCAT exon 4 160 96.0

Egr1 Rn00561138_m1 ACGAGCACCTGACCACAGAGTCCTT 1 - 2 exon junction 64 98.0

**_________________________________________________________________________________________________________________________________**

*taken from Applied Biosystems web-site.

**determined from Liu et al. Brain Research 1067(1), 1-12 (2006)
