# Supplementary material for: Transcriptional response of rat frontal cortex following acute In Vivo exposure to the pyrethroid insecticides permethrin and deltamethrin
Source: BMC Genomics. 2008 Nov 18;9:546. doi: 10.1186/1471-2164-9-546 (PMC2626604; doi:10.1186/1471-2164-9-546)
Supplement: Additional file 3 — List of probe sets with dose-dependent changes in expression for deltamethrin. Affymetrix probe set IDs without a gene symbol are expressed sequence tags (ESTs). Probe sets with arrows correspond to genes examined by qRT-PCR. Positive SAM di or PIR Mi scores denote upregulated probe sets. Negative SAM di or PIR Mi scores denote downregulated probe sets. [file 1471-2164-9-546-S3.doc]

**Additional File 3**. ***List of probe sets with dose-dependent changes in expression for deltamethrin.*** Affymetrix probe set IDs without a gene symbol are expressed sequence tags (ESTs). Probe sets with arrows correspond to genes examined by qRT-PCR. Positive SAM *d_i_* or PIR *M_i_* scores denote upregulated probe sets. Negative SAM *d_i_* or PIR *M_i_* scores denote downregulated probe sets.

|  | **Affymetrix** |  |  | **Linear Regression (SAM)** | | | **Isotonic Regression (PIR)** | | | **ANOVA** |
| --- | --- | --- | --- | --- | --- | --- | --- | --- | --- | --- |
|  | **Gene ID** | **GenBank** | **Gene Symbol** | **Score(*d_i_*)** | ***p*-value** | ***q*-value** | ***M_i_*** | ***p*-value** | ***q*-value** | ***p*-value** |
| **🡆** | 1371363_at | BI277042 | Gpd1 | 5.63 | 0.0000 | 0.00 | 2.34 | 0.0000 | 0.24 | 0.0032 |
| **🡆** | 1388901_at | AW534837 | Fkpb51 | 5.46 | 0.0000 | 0.00 | 2.21 | 0.0000 | 0.42 | 0.0051 |
| **🡆** | 1367577_at | NM_031970 | Hsp27 | 4.60 | 0.0001 | 0.00 | 1.90 | 0.0002 | 0.97 | 0.0195 |
| **🡆** | 1369560_at | NM_022215 | Gpd1 | 4.53 | 0.0001 | 0.00 | 1.91 | 0.0002 | 1.00 | 0.0134 |
|  | 1373415_at | AI407050 |  | 4.37 | 0.0001 | 0.00 | 1.80 | 0.0004 | 1.00 | 0.0195 |
| **🡆** | 1368064_a_at | U31884 | Ddc | -4.35 | 0.0001 | 0.14 | -1.95 | 0.0002 | 1.00 | 0.0195 |
| **🡆** | 1391229_at | BG381458 | Camk1g | 4.22 | 0.0002 | 0.00 | 2.11 | 0.0000 | 0.51 | 0.0063 |
|  | 1393128_at | BI288424 | RGD1311086 | 4.13 | 0.0002 | 0.00 | 1.76 | 0.0005 | 1.00 | 0.0205 |
| **🡆** | 1380611_at | BI284255 | Fkpb51 | 3.90 | 0.0004 | 0.11 | 1.62 | 0.0012 | 1.00 | 0.0276 |
|  | 1390659_at | BI302830 |  | 3.82 | 0.0005 | 0.11 | 1.49 | 0.0028 | 1.00 | 0.0276 |
|  | 1392492_at | AA956982 |  | 3.70 | 0.0007 | 0.11 | 1.66 | 0.0009 | 1.00 | 0.0276 |
|  | 1369303_at | NM_031019 | Crh | 3.62 | 0.0009 | 0.11 | 1.73 | 0.0006 | 1.00 | 0.0276 |
|  | 1389159_at | BM385437 |  | 3.56 | 0.0011 | 0.18 | 1.47 | 0.0031 | 1.00 | 0.0319 |
|  | 1375199_at | BG378641 |  | 3.54 | 0.0011 | 0.18 | 1.41 | 0.0045 | 1.00 | 0.0295 |
|  | 1390449_at | BI289132 |  | 3.48 | 0.0013 | 0.19 | 1.64 | 0.0011 | 1.00 | 0.0295 |
|  | 1388271_at | BM383531 | LOC689415 | 3.48 | 0.0013 | 0.19 | 1.44 | 0.0037 | 1.00 | 0.0300 |
|  | 1384396_at | AI144852 |  | 3.45 | 0.0014 | 0.19 | 1.62 | 0.0012 | 1.00 | 0.0276 |
|  | 1390606_at | BI289052 | RGD1564108_predicted | -3.42 | 0.0015 | 0.14 | -1.82 | 0.0004 | 1.00 | 0.0276 |
|  | 1382619_at | AI072460 |  | 3.39 | 0.0017 | 0.19 | 1.60 | 0.0013 | 1.00 | 0.0276 |
|  | 1370989_at | AI639318 | Ret | 3.38 | 0.0017 | 0.19 | 1.49 | 0.0028 | 1.00 | 0.0300 |
|  | 1391805_at | BE096676 | RGD1310364_predicted | 3.29 | 0.0022 | 0.29 | 1.50 | 0.0027 | 1.00 | 0.0300 |
|  | 1370026_at | NM_012935 | Cryab | 3.28 | 0.0022 | 0.29 | 1.46 | 0.0032 | 1.00 | 0.0364 |
|  | 1381693_at | AW526413 |  | -3.24 | 0.0024 | 0.14 | -1.39 | 0.0046 | 1.00 | 0.0364 |
|  | 1374626_at | BG371585 | Lrg1 | 3.23 | 0.0025 | 0.33 | 1.45 | 0.0036 | 1.00 | 0.0323 |
|  | 1378261_at | BE102806 |  | 3.15 | 0.0030 | 0.41 | 1.29 | 0.0089 | 1.00 | 0.0429 |
|  | 1373354_at | BF418347 |  | 3.14 | 0.0031 | 0.41 | 1.51 | 0.0025 | 1.00 | 0.0299 |
|  | 1372761_at | AI228076 |  | 3.09 | 0.0035 | 0.41 | 1.29 | 0.0088 | 1.00 | 0.0467 |
|  | 1378008_at | BF417386 |  | 3.08 | 0.0037 | 0.51 | 1.60 | 0.0014 | 1.00 | 0.0276 |
|  | 1376709_at | BM388442 | Slc39a8 | -3.07 | 0.0038 | 0.14 | -1.49 | 0.0026 | 1.00 | 0.0364 |
|  | 1368650_at | NM_031135 | Klf10 | -3.07 | 0.0038 | 0.14 | -1.30 | 0.0077 | 1.00 | 0.0356 |
|  | 1368891_at | AI014001 |  | 3.03 | 0.0041 | 0.51 | 1.34 | 0.0066 | 1.00 | 0.0460 |
|  | 1380329_at | AI717253 | Tmem10 | -3.03 | 0.0041 | 0.14 | -1.28 | 0.0086 | 1.00 | 0.0496 |
|  | 1373298_at | BI288011 |  | 3.03 | 0.0042 | 0.51 | 1.49 | 0.0027 | 1.00 | 0.0351 |
|  | 1376928_at | BE106737 |  | 3.03 | 0.0042 | 0.51 | 1.41 | 0.0045 | 1.00 | 0.0295 |
|  | 1372564_at | AI411375 | Ets2 | 3.03 | 0.0042 | 0.51 | 1.41 | 0.0045 | 1.00 | 0.0396 |
|  | 1382188_at | BF397703 | RGD1311086 | 3.02 | 0.0042 | 0.51 | 1.40 | 0.0048 | 1.00 | 0.0345 |
|  | 1380682_at | BF396302 | Rkhd3_predicted | 2.99 | 0.0045 | 0.51 | 1.45 | 0.0035 | 1.00 | 0.0329 |
|  | 1370454_at | AB003726 |  | 2.99 | 0.0046 | 0.51 | 1.32 | 0.0075 | 1.00 | 0.0369 |
|  | 1388522_at | AI170820 | RGD1310383_predicted | 2.97 | 0.0048 | 0.51 | 1.40 | 0.0047 | 1.00 | 0.0356 |
|  | 1389507_at | AI072446 | Nedd4l | 2.97 | 0.0048 | 0.51 | 1.42 | 0.0043 | 1.00 | 0.0306 |
|  | 1372491_at | AI229647 | RGD1565591_predicted | 2.92 | 0.0054 | 0.56 | 1.19 | 0.0159 | 1.00 | 0.0442 |
|  | 1375138_at | AA893169 | Timp3 | 2.92 | 0.0054 | 0.56 | 1.22 | 0.0134 | 1.00 | 0.0319 |
|  | 1371922_at | AI169140 |  | 2.91 | 0.0056 | 0.56 | 1.28 | 0.0098 | 1.00 | 0.0402 |
|  | 1374419_at | AI044435 |  | 2.90 | 0.0057 | 0.56 | 1.53 | 0.0021 | 1.00 | 0.0306 |
|  | 1372966_at | AI178784 | RGD1310174_predicted | 2.89 | 0.0059 | 0.56 | 1.23 | 0.0128 | 1.00 | 0.0476 |
|  | 1383665_at | BE096055 | Lpin2_predicted | 2.86 | 0.0063 | 0.59 | 1.21 | 0.0142 | 1.00 | 0.0496 |
|  | 1384841_at | AA858815 |  | -2.86 | 0.0064 | 0.14 | -1.46 | 0.0031 | 1.00 | 0.0421 |
|  | 1385892_at | AA900322 |  | 2.84 | 0.0067 | 1.15 | 1.40 | 0.0046 | 1.00 | 0.0276 |
|  | 1390163_at | BF282174 |  | 2.83 | 0.0068 | 1.15 | 1.38 | 0.0052 | 1.00 | 0.0440 |
|  | 1370530_a_at | AB000779 | Pld1 | -2.81 | 0.0071 | 0.14 | -1.34 | 0.0062 | 1.00 | 0.0493 |
|  | 1385778_at | BF409913 | Siat7E | 2.79 | 0.0075 | 1.15 | 1.38 | 0.0055 | 1.00 | 0.0276 |
|  | 1388401_at | BI296155 | Finb_predicted | 2.79 | 0.0076 | 1.15 | 1.17 | 0.0180 | 1.00 | 0.0429 |
|  | 1395986_at | BF391439 | Slit2 | -2.78 | 0.0077 | 0.14 | -1.27 | 0.0094 | 1.00 | 0.0419 |
|  | 1382186_a_at | AI136314 | RGD1311086 | 2.77 | 0.0078 | 1.15 | 1.15 | 0.0204 | 1.00 | 0.0442 |
|  | 1369973_at | NM_017154 | Xdh | 2.76 | 0.0080 | 1.15 | 1.18 | 0.0167 | 1.00 | 0.0472 |
|  | 1393337_at | AW524476 | Tcfcp2l1_predicted | 2.73 | 0.0088 | 1.16 | 1.37 | 0.0056 | 1.00 | 0.0276 |
|  | 1382225_at | BF284510 |  | 2.72 | 0.0090 | 1.16 | 1.28 | 0.0095 | 1.00 | 0.0427 |
|  | 1368438_at | NM_022236 | Pde10a | 2.70 | 0.0094 | 1.16 | 1.11 | 0.0255 | 1.00 | 0.0496 |
|  | 1387260_at | NM_053713 | Klf4 | -2.69 | 0.0096 | 0.14 | -1.49 | 0.0026 | 1.00 | 0.0429 |
|  | 1372356_at | BI285307 | Usp54 | 2.69 | 0.0097 | 1.16 | 1.12 | 0.0240 | 1.00 | 0.0442 |
|  | 1371442_at | BI282904 | Hyou1 | 2.68 | 0.0099 | 1.16 | 1.03 | 0.0384 | 1.00 | 0.0467 |
|  | 1375296_at | AI407178 | LOC684097 | 2.66 | 0.0105 | 1.16 | 1.40 | 0.0047 | 1.00 | 0.0427 |
|  | 1398899_at | AI170414 | Polr2c | 2.64 | 0.0110 | 1.16 | 1.67 | 0.0009 | 1.00 | 0.0195 |
|  | 1380835_at | BF389476 | RGD1565346_predicted | 2.62 | 0.0115 | 1.16 | 1.38 | 0.0053 | 1.00 | 0.0467 |
| **🡆** | 1377518_at | AW251224 | Camk1g | 2.59 | 0.0123 | 1.16 | 1.29 | 0.0090 | 1.00 | 0.0315 |
|  | 1375761_at | AW532391 |  | 2.57 | 0.0128 | 1.16 | 1.36 | 0.0061 | 1.00 | 0.0356 |
|  | 1383861_at | BF394135 |  | 2.57 | 0.0129 | 1.16 | 1.35 | 0.0065 | 1.00 | 0.0306 |
|  | 1392321_at | BE120641 |  | 2.57 | 0.0130 | 1.16 | 1.29 | 0.0092 | 1.00 | 0.0467 |
|  | 1372090_at | AI231566 | Max | 2.53 | 0.0143 | 1.16 | 1.31 | 0.0080 | 1.00 | 0.0306 |
|  | 1397261_at | AI547508 |  | -2.50 | 0.0152 | 0.14 | -1.42 | 0.0040 | 1.00 | 0.0295 |
|  | 1376768_at | BM386807 |  | 2.40 | 0.0191 | 1.16 | 1.52 | 0.0023 | 1.00 | 0.0276 |
|  | 1381557_at | BI289045 | Gna14 | 2.35 | 0.0216 | 1.16 | 1.29 | 0.0087 | 1.00 | 0.0442 |
|  | 1397677_at | AI501069 |  | -2.30 | 0.0243 | 0.14 | -1.42 | 0.0039 | 1.00 | 0.0467 |
|  | 1398373_at | AA799400 | B3galt3 | 2.29 | 0.0248 | 1.16 | 1.29 | 0.0091 | 1.00 | 0.0396 |
|  | 1395253_at | BE107893 |  | -2.25 | 0.0273 | 0.14 | -1.39 | 0.0046 | 1.00 | 0.0388 |
|  | 1372037_at | AI104117 | Pdlm7 | 2.18 | 0.0319 | 1.16 | 1.28 | 0.0098 | 1.00 | 0.0351 |
|  | 1382112_at | BM385698 | LOC682926 | -2.16 | 0.0333 | 1.15 | -1.26 | 0.0096 | 1.00 | 0.0344 |
|  | 1397229_at | BF565781 |  | -2.15 | 0.0342 | 1.15 | -1.26 | 0.0095 | 1.00 | 0.0295 |
|  | 1391147_at | BF404398 |  | -2.13 | 0.0354 | 1.15 | -1.36 | 0.0056 | 1.00 | 0.0427 |
|  | 1397198_at | BE111113 |  | -2.13 | 0.0361 | 1.15 | -1.26 | 0.0096 | 1.00 | 0.0427 |
|  | 1396401_at | AW433899 |  | 2.11 | 0.0377 | 1.16 | 1.47 | 0.0031 | 1.00 | 0.0319 |
|  | 1385821_at | BF392004 |  | -2.09 | 0.0390 | 1.15 | -1.55 | 0.0019 | 1.00 | 0.0300 |
|  | 1384959_at | BI295935 |  | 2.03 | 0.0449 | 1.16 | 1.41 | 0.0045 | 1.00 | 0.0295 |
|  | 1372448_at | D86711 | Medl19_predicted | 2.01 | 0.0467 | 1.16 | 1.36 | 0.0060 | 1.00 | 0.0276 |
|  | 1376463_at | AA955579 |  | -1.97 | 0.0510 | 1.15 | -1.33 | 0.0067 | 1.00 | 0.0402 |
|  | 1386344_at | BG662519 |  | 1.97 | 0.0511 | 1.16 | 1.29 | 0.0089 | 1.00 | 0.0295 |
|  | 1395169_at | BF388779 | Zcch8_predicted | 1.96 | 0.0516 | 1.16 | 1.29 | 0.0092 | 1.00 | 0.0442 |
|  | 1375752_at | AI577874 | Bves | -1.96 | 0.0519 | 1.15 | -1.41 | 0.0042 | 1.00 | 0.0376 |
|  | 1370869_at | AI102790 | Bcat1 | 1.92 | 0.0561 | 1.16 | 1.28 | 0.0096 | 1.00 | 0.0345 |
|  | 1367706_at | NM_031353 | Vdac1 | 1.74 | 0.0820 | 1.16 | 1.29 | 0.0090 | 1.00 | 0.0295 |
|  | 1385645_at | AA875088 |  | -1.73 | 0.0827 | 1.15 | -1.32 | 0.0069 | 1.00 | 0.0295 |
|  | 1377514_at | BF413478 |  | -1.59 | 0.1087 | 1.15 | -1.30 | 0.0079 | 1.00 | 0.0295 |
|  | 1380905_at | AA893260 |  | -1.56 | 0.1162 | 1.15 | -1.72 | 0.0007 | 1.00 | 0.0276 |
|  | 1393978_at | BF415134 | Stfa2_predicted | 1.40 | 0.1564 | 1.16 | 1.32 | 0.0077 | 1.00 | 0.0223 |
|  | 1396505_at | BE113909 |  | -0.64 | 0.5102 | 1.15 | -1.27 | 0.0090 | 1.00 | 0.0254 |
