# Supplementary material for: Transcriptional response of rat frontal cortex following acute In Vivo exposure to the pyrethroid insecticides permethrin and deltamethrin
Source: BMC Genomics. 2008 Nov 18;9:546. doi: 10.1186/1471-2164-9-546 (PMC2626604; doi:10.1186/1471-2164-9-546)
Supplement: Additional file 4 — List of probe sets with dose-dependent changes in expression for permethrin. Affymetrix probe set IDs without a gene symbol are expressed sequence tags (ESTs). Probe sets with arrows correspond to genes examined by qRT-PCR. Positive SAM di or PIR Mi scores denote upregulated probe sets. Negative SAM di or PIR Mi scores denote downregulated probe sets. [file 1471-2164-9-546-S4.doc]

**Additional File 4**. ***List of probe sets with dose-dependent changes in expression for permethrin.*** Affymetrix probe set IDs without a gene symbol are expressed sequence tags (ESTs). Probe sets with arrows correspond to genes examined by qRT-PCR. Positive SAM *d_i_* or PIR *M_i_* scores denote upregulated probe sets. Negative SAM *d_i_* or PIR *M_i_* scores denote downregulated probe sets.

|  | **Affymetrix** |  |  | **Linear Regression (SAM)** | | | **Isotonic Regression (PIR)** | | | **ANOVA** |
| --- | --- | --- | --- | --- | --- | --- | --- | --- | --- | --- |
|  | **Gene ID** | **GenBank** | **Gene Symbol** | **Score(*d_i_*)** | ***p*-value** | ***q*-value** | ***M_i_*** | ***p*-value** | ***q*-value** | ***p*-value** |
|  | 1393119_at | BM388725 |  | 5.2 | 0 | 0 | 2.22 | 0 | 0.96 | 0.005 |
|  | 1373415_at | AI407050 |  | 4.89 | 0.0001 | 0 | 2.06 | 0.0001 | 1 | 0.005 |
|  | 1373035_at | AI031032 |  | 4.76 | 0.0001 | 0 | 1.96 | 0.0002 | 1 | 0.0078 |
|  | 1373298_at | BI288011 |  | 4.22 | 0.0001 | 0 | 2.08 | 0.0001 | 1 | 0.0078 |
|  | 1369303_at | NM_031019 | Crh | 4.26 | 0.0001 | 0 | 1.92 | 0.0003 | 1 | 0.0113 |
|  | 1391901_at | AA956085 |  | 4.19 | 0.0002 | 0 | 1.84 | 0.0005 | 1 | 0.0139 |
|  | 1374610_at | AI599365 |  | 4.06 | 0.0002 | 0 | 1.91 | 0.0003 | 1 | 0.0139 |
|  | 1392791_at | AA964492 |  | 3.88 | 0.0003 | 0 | 1.65 | 0.0012 | 1 | 0.017 |
| 🡆 | 1368677_at | NM_012513 | Bdnf | 3.85 | 0.0003 | 0 | 1.52 | 0.0025 | 1 | 0.017 |
|  | 1370454_at | AB003726 |  | 3.85 | 0.0003 | 0 | 1.15 | 0.0185 | 1 | 0.0215 |
|  | 1395197_at | BI293027 |  | -3.61 | 0.0005 | 0.18 | -1.75 | 0.0013 | 1 | 0.023 |
|  | 1390412_at | AI229664 | Slc40a1 | -3.5 | 0.0006 | 0.18 | -1.7 | 0.0016 | 1 | 0.005 |
| 🡆 | 1370415_at | AF002251 | Rassf5 | 3.37 | 0.0008 | 0.12 | 1.85 | 0.0004 | 1 | 0.017 |
|  | 1382225_at | BF284510 |  | 3.29 | 0.001 | 0.12 | 1.5 | 0.0028 | 1 | 0.0349 |
|  | 1393389_at | BF396237 |  | 3.25 | 0.0011 | 0.18 | 1.39 | 0.0049 | 1 | 0.017 |
| 🡆 | 1375043_at | BF415939 | c-fos | 3.23 | 0.0012 | 0.18 | 1.41 | 0.0045 | 1 | 0.017 |
|  | 1371731_at | AI408151 | RGD1566215_predicted | 3.13 | 0.0015 | 0.28 | 1.44 | 0.0038 | 1 | 0.0315 |
|  | 1395991_at | BE107556 | Rimbp2 | 3.11 | 0.0016 | 0.28 | 1.39 | 0.005 | 1 | 0.04 |
|  | 1388583_at | BF283398 | Cxcl12 | 2.92 | 0.0027 | 0.42 | 1.36 | 0.0057 | 1 | 0.0477 |
| 🡆 | 1368321_at | NM_012551 | Egr1 | 2.87 | 0.003 | 0.5 | 1.21 | 0.0132 | 1 | 0.023 |
|  | 1392108_at | BF390648 |  | 2.85 | 0.0032 | 0.5 | 1.34 | 0.0064 | 1 | 0.0347 |
|  | 1375986_at | AI103155 |  | 2.83 | 0.0034 | 0.5 | 1.59 | 0.0017 | 1 | 0.023 |
|  | 1379910_at | AI136097 | RGD1561967_predicted | 2.79 | 0.0037 | 0.55 | 1.42 | 0.0043 | 1 | 0.017 |
|  | 1369067_at | NM_031628 | Nr4a3 | 2.77 | 0.004 | 0.55 | 1.15 | 0.0187 | 1 | 0.027 |
|  | 1387025_at | NM_019234 | Dync1i1 | 2.76 | 0.0041 | 0.55 | 1.29 | 0.0085 | 1 | 0.0208 |
|  | 1382613_at | AW144049 |  | -2.68 | 0.0051 | 0.69 | -1.5 | 0.0039 | 1 | 0.0408 |
|  | 1381557_at | BI289045 | Gna14 | 2.67 | 0.0052 | 0.61 | 1.87 | 0.0004 | 1 | 0.0176 |
|  | 1372019_at | AI231789 | RGD1310128_predicted | 2.62 | 0.0059 | 0.69 | 1.35 | 0.0061 | 1 | 0.023 |
|  | 1387024_at | NM_053883 | Dusp6 | 2.59 | 0.0064 | 0.75 | 1.01 | 0.0398 | 1 | 0.023 |
|  | 1398464_at | AI575255 |  | -2.58 | 0.0066 | 0.69 | -1.38 | 0.0067 | 1 | 0.017 |
|  | 1372363_at | BF404414 | RGD1561203_predicted | 2.54 | 0.0073 | 0.79 | 1.33 | 0.0067 | 1 | 0.023 |
|  | 1374787_at | BI282169 |  | 2.5 | 0.0081 | 0.79 | 1.34 | 0.0064 | 1 | 0.023 |
|  | 1388911_at | AI177134 | Prim2 | 2.5 | 0.0082 | 0.79 | 1.45 | 0.0036 | 1 | 0.0408 |
|  | 1372998_at | BG381555 |  | 2.45 | 0.0094 | 0.79 | 1.2 | 0.0144 | 1 | 0.0405 |
|  | 1376616_at | BF551036 |  | -2.4 | 0.0107 | 0.69 | -1.36 | 0.0071 | 1 | 0.0429 |
|  | 1385778_at | BF409913 | Siat7E | 2.36 | 0.0116 | 0.79 | 1.43 | 0.0041 | 1 | 0.0305 |
|  | 1396747_at | BE121159 |  | 2.33 | 0.0128 | 0.79 | 1.32 | 0.0071 | 1 | 0.0428 |
|  | 1378407_at | BF401415 |  | 2.28 | 0.0145 | 0.79 | 1.29 | 0.0083 | 1 | 0.0315 |
|  | 1382275_at | AI236989 | MGC125015 | 2.24 | 0.0158 | 0.79 | 1.46 | 0.0034 | 1 | 0.023 |
|  | 1381070_at | AI233106 |  | 2.01 | 0.0286 | 0.79 | 1.51 | 0.0026 | 1 | 0.0351 |
|  | 1395272_at | BF394456 | LOC682937 | -1.88 | 0.0394 | 0.69 | -1.33 | 0.0084 | 1 | 0.0305 |
|  | 1390716_at | BE098148 |  | -1.87 | 0.0401 | 0.69 | -1.55 | 0.0031 | 1 | 0.023 |
|  | 1383685_at | BI276972 | Heatr1_predicted | 1.54 | 0.0836 | 0.79 | 1.32 | 0.0072 | 1 | 0.023 |
|  | 1381400_at | AI137973 |  | 1.42 | 0.108 | 0.79 | 1.33 | 0.0068 | 1 | 0.023 |
|  | 1367652_at | AI713966 | Igfpb3 | 1.38 | 0.119 | 0.79 | 1.35 | 0.0063 | 1 | 0.0358 |
|  | 1380509_at | AW253985 |  | 1.37 | 0.1193 | 0.79 | 1.4 | 0.0047 | 1 | 0.023 |
|  | 1389090_at | BI284350 | Wrnip1 | 1.32 | 0.1319 | 0.79 | 1.3 | 0.0079 | 1 | 0.017 |
|  | 1384884_at | AW528484 | RGD1307595_predicted | 1.18 | 0.1755 | 1.32 | 1.35 | 0.006 | 1 | 0.0405 |
|  | 1397745_at | BF414336 |  | -0.91 | 0.286 | 0.69 | -1.43 | 0.0051 | 1 | 0.023 |
|  | 1376602_a_at | AI030899 | Fbxo22 | 0.89 | 0.2974 | 1.32 | 1.27 | 0.0096 | 1 | 0.0186 |
|  | 1395303_at | BF397734 |  | -0.8 | 0.3465 | 0.69 | -1.48 | 0.0041 | 1 | 0.0139 |
|  | 1396401_at | AW433899 |  | 0.71 | 0.4017 | 1.32 | 1.44 | 0.0038 | 1 | 0.0139 |
|  | 1391301_at | AA997499 | LOC682355 | 0.51 | 0.5404 | 1.32 | 1.35 | 0.0062 | 1 | 0.0305 |
