# Supplementary material for: Transcriptional response of rat frontal cortex following acute In Vivo exposure to the pyrethroid insecticides permethrin and deltamethrin
Source: BMC Genomics. 2008 Nov 18;9:546. doi: 10.1186/1471-2164-9-546 (PMC2626604; doi:10.1186/1471-2164-9-546)
Supplement: Additional file 5 — Two-way analysis of variance (ANOVA) for qRT-PCR time course data. [file 1471-2164-9-546-S5.doc]

## **Additional File 5. *Two-way analysis of variance (ANOVA) for qRT-PCR time course data.***

**3 mg/kg Deltamethrin 100 mg/kg Permethrin**

**Gene Pair Pair**

**Name Factor *F* *p*-value Wise *F* *p*-value Wise** ___________________________________________________________________

**Camk1g** TRT 4.56 0.0371** 3 h 2.16 0.1472 n.e.

TIME 1.75 0.1665 1.14 0.3391

TRT*TIME 2.07 0.1140 0.54 0.6586

**Gpd1** TRT 17.56 n/a 3,6 h 12.76 0.0007** 6 h

TIME 4.05 n/a 1.55 0.2126

TRT*TIME 4.44 0.0072** 1.42 0.2465

**Ddc** TRT 4.48 0.0387 6,9 h 0.01 0.9419 n.e.

TIME 1.78 0.1620 1.01 0.3970

TRT*TIME 2.17 0.1025 0.91 0.4438

**c-fos** TRT 5.78 n/a 3 h 10.66 0.0019** 3 h

TIME 6.35 n/a 2.99 0.0386**

TRT*TIME 6.26 0.0010** 1.56 0.2014

**Egr1** TRT 7.63 n/a 3 h 26.12 n/a 3,6 h

TIME 10.43 n/a 8.26 n/a

TRT*TIME 7.62 0.0002** 8.40 0.0001**

**BDNF** TRT 0.14 0.7110 n.e. 2.04 0.1587 n.e.

TIME 0.29 0.8358 1.24 0.3052

TRT*TIME 0.22 0.8814 0.69 0.5633

_____________________________________________________________________

Two-way ANOVA factors are: TIME, TRT, TIME*TRT. Analysis of 2^-ΔΔCT^ values is presented. n.e. = no effect. n/a = not applicable. ** = significant effect at *p* < 0.05. Pair-wise comparisons are within time for a main effect of treatment (*p* < 0.05).
